# Transcriptional downregulation of miR-133b by REST promotes prostate cancer metastasis to bone via activating TGF-β signaling

**DOI:** 10.1038/s41419-018-0807-3

**Published:** 2018-07-13

**Authors:** Shuai Huang, Qingde Wa, Jincheng Pan, Xinsheng Peng, Dong Ren, Qiji Li, Yuhu Dai, Qing Yang, Yan Huang, Xin Zhang, Wei Zhou, Dan Yuan, Jiazheng Cao, Yuming Li, Peiheng He, Yubo Tang

**Affiliations:** 1 Department of Orthopaedic Surgery, the Second Affiliated Hospital of Guangzhou Medical University, 510260 Guangzhou, China; 2 Department of Orthopaedic Surgery, the First Affiliated Hospital of Sun Yat-sen University, 510080 Guangzhou, China; 3 Department of Orthopaedic Surgery, the Affiliated Hospital of Zunyi Medical college, 563003 Zunyi, China; 4 Department of Urology Surgery, the First Affiliated Hospital of Sun Yat-sen University, 510080 Guangzhou, China; 5 Department of Pathology, Jiangmen Central Hospital, Affiliated Jiangmen Hospital of Sun Yat-sen University, Jiangmen, 529030 China; 6 Department of Urology, Jiangmen Central Hospital, Affiliated Jiangmen Hospital of Sun Yat-sen University, Jiangmen, 529030 China; 7 Department of Orthopaedic Surgery, Jiangmen Central Hospital, Affiliated Jiangmen Hospital of Sun Yat-sen University, Jiangmen, 529030 China; 8 Department of Pharmacy, The First Affiliated Hospital of Sun Yat-Sen University, 510080 Guangzhou, China

## Abstract

High avidity of bone metastasis is an important characteristic in prostate cancer (PCa). Downexpression of miR-133b has been reported to be implicated in the development, progression and recurrence in PCa. However, clinical significance and biological roles of miR-133b in bone metastasis of PCa remain unclear. Here we report that miR-133b is downregulated in PCa tissues and further decreased in bone metastatic PCa tissues. Downexpression of miR-133b positively correlates with advanced clinicopathological characteristics and shorter bone metastasis-free survival in PCa patients. Upregulating miR-133b inhibits invasion, migration in vitro and bone metastasis in vivo in PCa cells. Mechanistically, we find that miR-133b suppresses activity of TGF-β signaling via directly targeting TGF-β receptor I and II, which further inhibits bone metastasis of PCa cells. Our results further reveal that overexpression of REST contributes to miR-133b downexpression via transcriptional repression in PCa tissues. Importantly, silencing miR-133b enhances invasion and migration abilities in vitro and bone metastasis ability in vivo in REST-silenced PCa cells. The clinical correlation of miR-133b with TGFBRI, TGFBRII, REST and TGF-β signaling activity is verified in PCa tissues. Therefore, our results uncover a novel mechanism of miR-133b downexpression that REST transcriptionally inhibits miR-133b expression in PCa cells, and meanwhile support the notion that administration of miR-133b may serve as a rational regimen in the treatment of PCa bone metastasis.

## Introduction

Prostate cancer (PCa) is the second most frequently diagnosed cancer among men and the fifth leading cause of cancer-related deaths worldwide^1^. Despite mortality rate of PCa patients has been improved in the majority of developed countries, it remains poor, even rising in some developing countries, including China^2^. Tumor cell metastasis to distant sites, particularly bone, greatly contributes to high mortality of PCa patients^3,4^. Bone is among the most preferential metastatic site of PCa^5^. Once bone metastasis happens, which brings numerous bone- associated complications, including hypercalcemia, intractable pain, fracture, or nerve compression syndrome, causing poor survival^6^.Therefore, understanding the molecular mechanism underlying the metastatic proclivity of PCa cells to bone is a profound task that may facilitate the development of strategies to prevent or treat bone metastasis of PCa.

TGF-β signaling has been demonstrated to play an important role in several biological processes, including cell differentiation, embryogenesis and bone remodeling^7^. From a molecular perspective, TGF-β signaling starts via ligand-induced cooperative assembly of the receptor complex, where the kinase domain of the type II receptor phosphorylates the type I receptor and receptor-bound SMAD (R-Smad) proteins 2 and 3, which further drives assembly of R-Smads into a complex with a second class of Smads, the so-called co-Smad (Smad4), leading to the nuclear accumulation of heteromeric Smad complexes^8^. In the nucleus, Smads regulate transcription of TGF-β target genes as transcriptional factors by interacting with a broad range of DNA-binding partners^9^. In cancer, TGF-β signaling functions as either an oncogenic or tumor-suppressive pathway depending on the developmental stage and type of tumor: in early stages, TGF-β signaling inhibits cell growth as a tumor suppressor, while in later stage of cancer, TGF-β promotes invasion and metastasis, in particular metastasis to bone^10^. TGF-β signaling promote bone metastasis of cancers via transcriptionally regulating multiple bone metastasis-associated genes, including MMP1, IL-11, or PTHRP^11–13^. Accumulating literatures have reported that TGF-β signaling plays a pivotal role in the development of bone metastasis in cancer, including breast cancer and melanoma^14,15^. Importantly, the pro-bone metastasis role of TGF-β signaling in PCa has been demonstrated. Fournier and colleagues reported that activation of TGF-β signaling upregulated PMEPA1, an important negative regulator of the TGF-β pathway, and that interrupting this negative feedback loop by PMEPA1 knockdown drove PCa cells to disseminate to bone marrow, ultimately increasing bone metastases in a mouse PCa model^16^. Furthermore, therapy targeting TGF-β significantly reduced the development of bone metastases in PCa^17,18^. However, the underlying molecular mechanisms responsible for constitutive activation of TGF-β in PCa bone metastasis have not been determined.

Since its discovery, RE1-silencing transcription factor (REST) has been identified to act as a transcriptional repressor typically expressed in non-neuronal tissues, where it suppresses the expression of neuronal genes, and plays an important role in neuronal development^19,20^. Recently, mutations or overexpression of REST have been extensively reported in various types of cancers^21–23^. Strikingly, Kreisler and colleagues have demonstrated that hypermethylation induced loss of REST expression in small-cell lung cancer, which was linked to malignant progression of small-cell lung cancer via leading to an epidermal growth factor-mediated de-regulation of AKT-Serine473 phosphorylation^24^. These studies indicate that REST may act as an oncogene or tumor suppressor dependent on tumor type. In PCa, loss of REST has been reported to be involved in neuroendocrine differentiation^25,26^. However, no data is available for the role of REST alterations in bone metastasis of PCa.

microRNAs (miRNAs) are short, noncoding RNAs that regulate downstream target genes expression at a post-transcriptional level via binding with specific sequences in the 3′ untranslated region (3′-UTR) of downstream target genes^27^. miRNAs play important roles in many cellular and biological processes^28^, and aberrant expression of miRNAs have been reported to be implicated in the progression and metastasis of cancers^29–34^. Furthermore, several miRNAs have been reported as critical mediators in the bone metastasis of PCa^35–37^. Our previous studies demonstrated that the loss of wild-type p53 induced downexpression of miR-145, promoted bone metastasis of PCa via regulating several positive regulators of EMT^38–40^. These studies indicate that aberrant expression of miRNAs is significantly associated with the bone metastasis of PCa.

In this study, our results found that miR-133b was downregulated in PCa tissues and further decreased in bone metastatic PCa tissues, and miR-133b expression levels was inversely associated with poor clinicopathological characteristics and bone metastasis-free survival in PCa patients. In addition, upregulating miR-133b dramatically inhibited invasion and migration in vitro, and bone metastasis in vivo in PCa cells. Our results further demonstrated that miR-133b directly targeted TGFBRI and TGFBRII, which further repressed activity of TGF-β signaling, invasion and migration abilities of PCa cells. Importantly, our findings revealed that REST transcriptionally inhibited miR-133b expression, leading to miR-133b downexpression in PCa tissues. Furthermore, downregulating miR-133b reversed the effects of silencing REST on bone metastasis of PCa in vitro and in vivo. Therefore, our results indicate that REST/miR-133b/TGF-β signaling axis plays an important role in bone metastasis of PCa.

## Results

### miR-133b is downregulated in PCa tissues and further decreased in bone metastatic PCa tissues

**Fig. 1:**
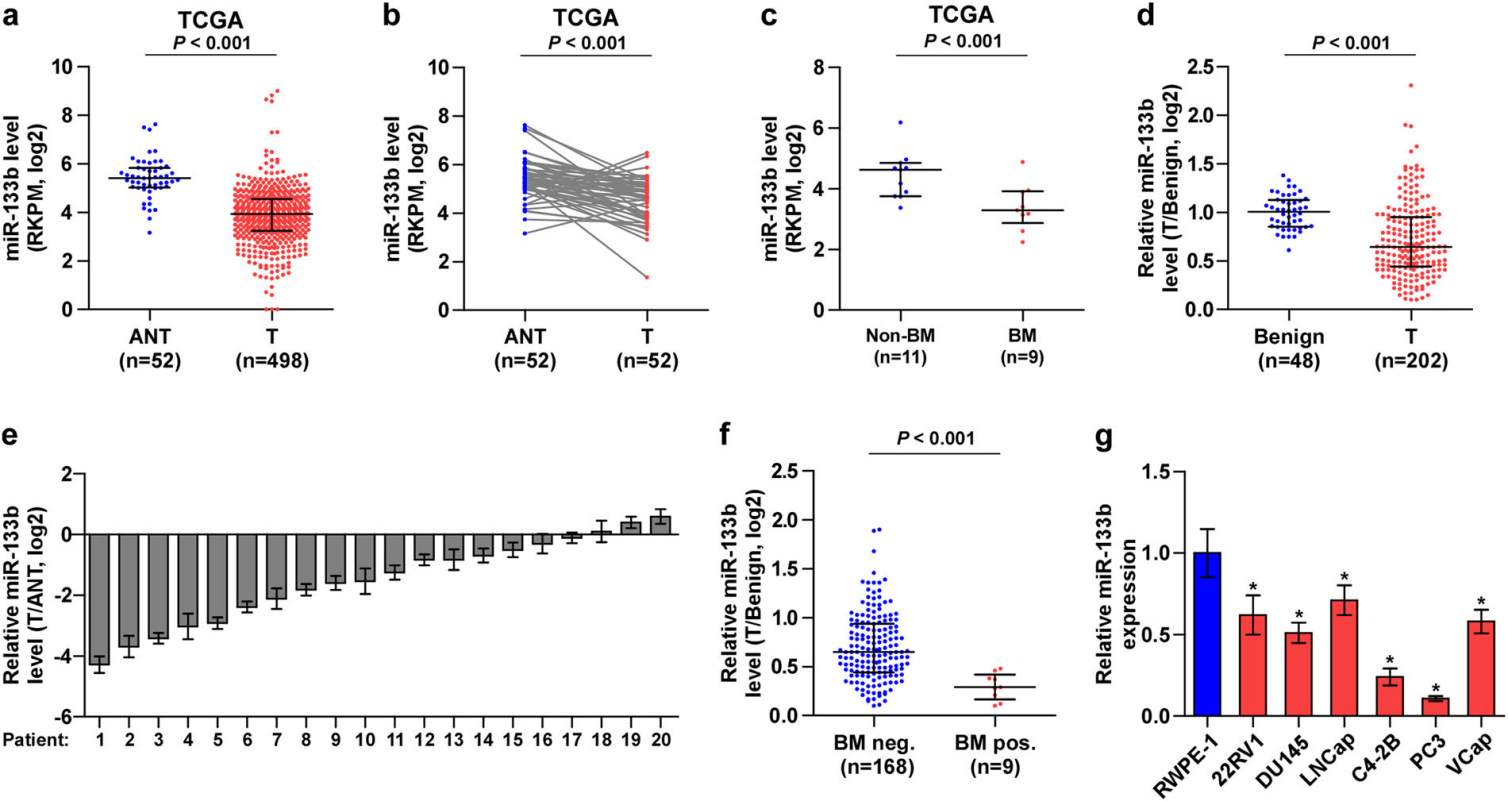
miR-133b is downregulated in PCa tissues and further decreased in bone metastatic PCa tissues. **a** miR-133b expression levels was decreased in PCa tissues compared with that in adjacent normal tissues (ANT) by analyzing the PCa miRNA sequencing data set from TCGA (ANT, *n* = 52; PCa, *n* = 498). **b** miR-133b expression levels was reduced in 52 paired PCa tissues compared with that in the matching ANT by analyzing the PCa miRNA sequencing data set from TCGA. **c** miR-133b expression levels was further downregulated in bone metastatic PCa tissues (BM) compared with that in non-bone metastatic PCa tissues (non-BM) by analyzing the PCa miRNA sequencing data set from TCGA. (non-BM, *n* = 11; BM, *n* = 9). **d** Real-time PCR analysis of miR-133b expression in 48 benign prostate lesions tissues and 202 PCa tissues. Transcript levels were normalized to *U6* expression. Lines represent median and lower/upper quartiles. **e** Real-time PCR analysis of miR-133b expression ration in 20 paired PCa tissues (miR-133b expression level in PCa tissues: miR-133b expression level in benign tissues). Transcript levels were normalized to *U6* expression. **f** Real-time PCR analysis of miR-133b expression in 168 non-bone metastatic and 9 bone metastatic PCa samples. Transcript levels were normalized to *U6* expression. Lines represent median and lower/upper quartiles. *P *<* 0.05. **g** Real-time PCR analysis of miR-133b expression levels in normal prostate epithelial cell (RWPE-1), primary PCa cell 22RV1, bone metastatic PCa cell lines (PC-3, C4-2B and VCaP) and brain metastatic cell line DU145 and lymph node metastatic cell line LNCaP. Transcript levels were normalized to *U6* expression. *P *<* 0.05

To investigate the clinical significance of miR-133b in PCa, we first analyzed several publicly available miRNA sequencing data set of PCa from The Cancer Genome Atlas (TCGA) and ArrayExpress, and found that miR-133b expression was downregulated in separate and paired PCa tissues compared with the adjacent normal tissues (ANT) (Fig. 1a, b, and Supplementary Figure 1a and 1b), and further reduced in bone metastatic PCa tissues compared with that in non-bone metastatic PCa tissues (Fig. 1c). Consistently, low expression of miR-133b was demonstrated in our 202 individual and 20 paired PCa tissues compared with benign prostate lesions, including benign prostate hyperplasia and prostatitis (Fig. 1d,e), particularly in bone metastatic PCa tissues (Fig. 1f). We further examined the expression levels of miR-133b in normal prostate epithelial cells RWPE-1 and other 6 PCa cells and found that miR-133b expression were differentially decreased compared with RWPE-1, especially in bone metastatic PCa cell lines (PC-3 and C4-2B) (Fig. 1g). These results indicate that downexpression of miR-133b may be associated with the progression and bone metastasis of PCa.

### Downexpression of miR-133b correlates with advanced clinicopathological characteristics and poor bone metastasis-free survival in PCa patients

**Fig. 2:**
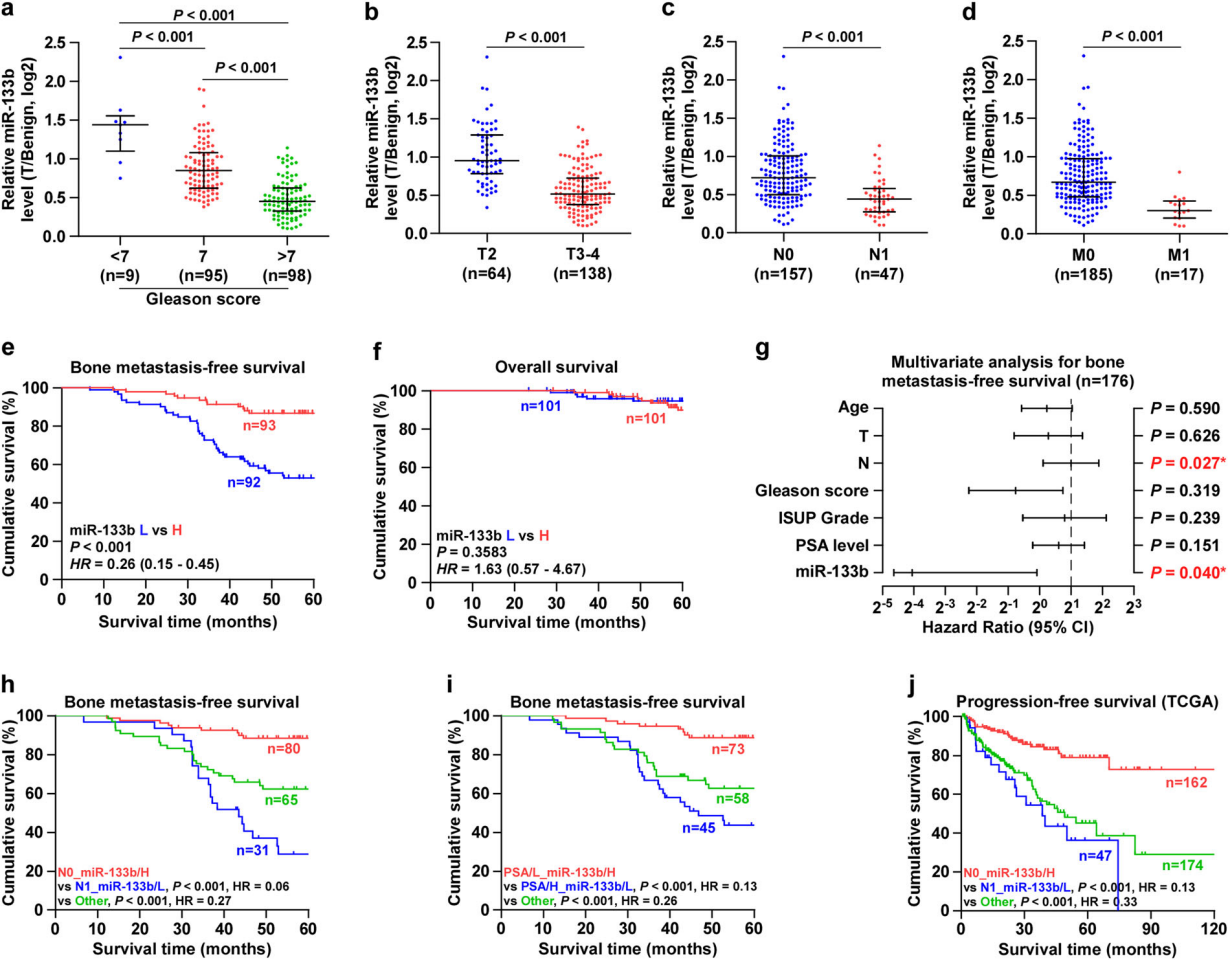
Low expression of miR-133b correlates with poor clinicopathological characteristics and bone metastasis-free survival in PCa patients. **a** miR-133b expression levels in PCa tissues with different Gleason score. **b** miR-133b expression levels in PCa tissues with different tumor volume. **c** miR-133b expression levels in PCa tissues with different lymph node metastasis status. **d** miR-133b expression levels in PCa tissues with different distant metastasis status. **e** Kaplan–Meier analysis of bone metastasis-free survival curves of PCa patients with high miR-133b expression (*n* = 93) vs. low miR-133b expression (*n* = 92). The mean survival times: miR-133b-low > 60 months, and miR-133b-high > 60 months. **f** Kaplan–Meier analysis of overall survival curves of PCa patients with high miR-133b expression (*n* = 101) vs. low miR-133b expression (*n* = 101). The mean survival times: miR-133b-low > 60 months, and miR-133b-high > 60 months. **g** Multivariate Cox regression analysis to evaluate the significance of the association between miR-133b expression and bone metastasis-free survival. HR values were presented by log2 transformation. **h** Kaplan–Meier analysis of bone metastasis-free survival curves of the PCa patients stratified by miR-133b expression and lymph node metastasis status. The mean survival times: N0_miR-133b-high > 60 months, N1_miR-133b-low = 43.5 months, and other > 60 months. **i** Kaplan–Meier analysis of bone metastasis-free survival curves of the PCa patients stratified by miR-133b expression and serum PSA levels. The mean survival times: PSA/L_miR-133b-high > 60 months, PSA/H_miR-133b-low = 46.8 months, and other > 60 months. **j** Kaplan–Meier analysis of progression-free survival curves of the PCa patients stratified by miR-133b expression and lymph node metastasis status as assessed by TCGA. The mean survival times: N0_miR-133b-high > 120 months, N1_miR-133b-low = 38.5 months, and other = 49.1 months

We further analyzed the clinical correlation of miR-133b expression levels with clinicopathological characteristics in PCa. As shown in Fig. 2a–d, Supplementary Figure 2a–d and Supplementary Table 1, low expression of miR-133b positively correlated with Gleason grade, T classification, N classification and M classification in PCa patients. Kaplan–Meier survival analysis indicated that PCa patients with low miR-133b expression correlated with shorter bone metastasis-free and progression-free survival (Fig. 2e and Supplementary Figure 2f). Univariate Cox-regression analysis indicated patients with low miR-133b expression had shorter bone metastasis-free survivals (*P* < 0.001; hazard ratio = 0.08, 95% CI = 0.03–0.22) compared to patients with high miR-133b expression (Supplementary Table 2). Multivariate Cox regression analysis revealed that low expression of miR-133b may be used as independent factors to predict bone metastasis-free survival (Fig. 2g and Supplementary Table 2). However, low expression of miR-133b had no obvious effect on overall survival in PCa patients (Fig. 2f, Supplementary Figure 2e and Supplementary Table 3). It was noteworthy that downexpression of miR-133b combined with lymph node metastasis or high level of serum PSA predicted poorer bone metastasis-free and progression-free survival (Fig. 2h–j). Taken together, our results indicated that low expression of miR-133b is positively associated with poor bone metastasis-free and progression-free survival in PCa patients.

### miR-133b inhibits TGF-β signaling pathway

**Fig. 3:**
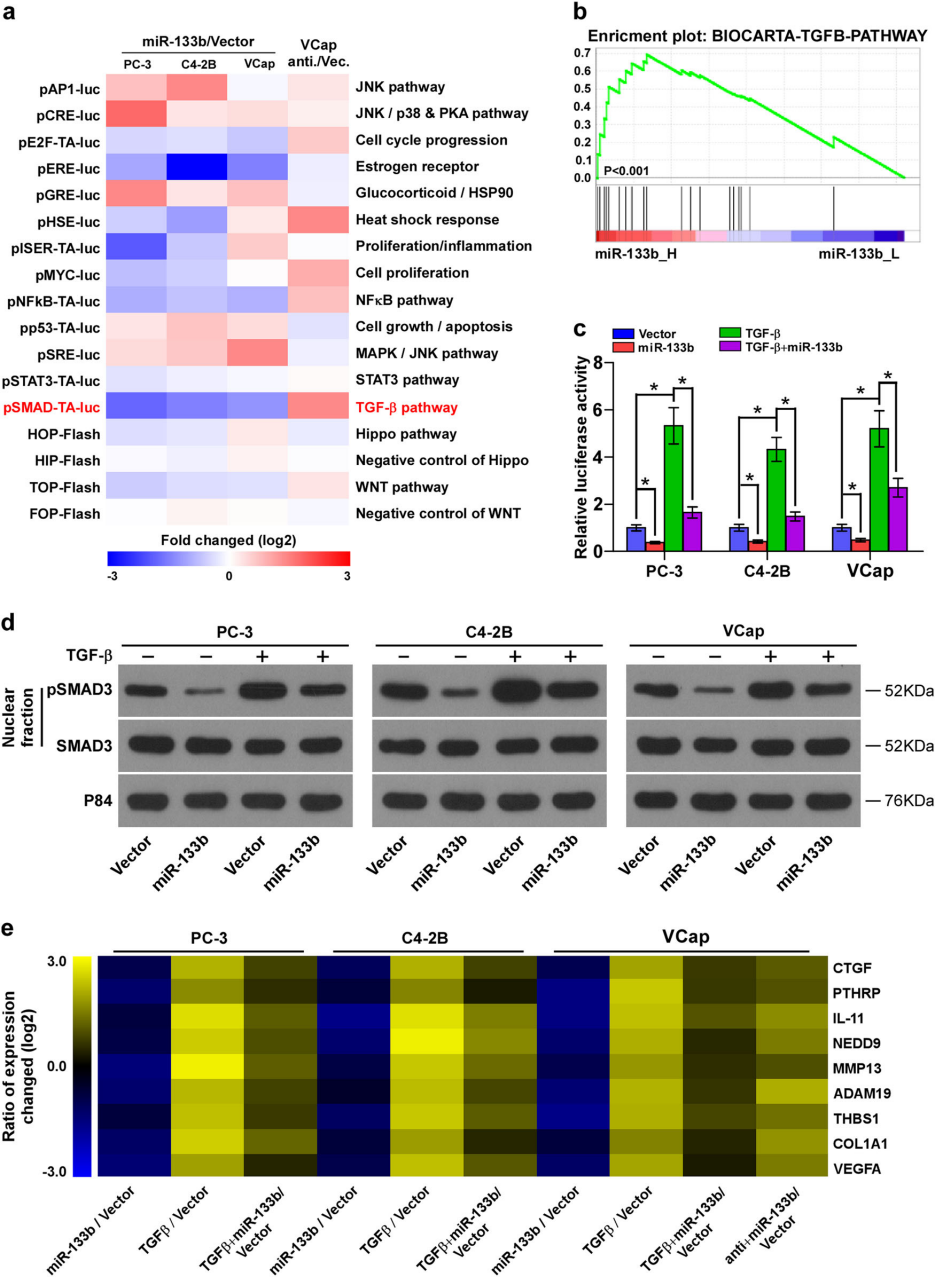
Overexpression of miR-133b inhibits TGF-β signaling activity in PCa cells. **a** Activity of luciferase reporter constructs of several signaling pathway were examined in the miR-133b-overexpressing or –silencing PCa cells. **b** Gene set enrichment analysis (GSEA) revealed that miR-133b expression correlated with TGF-β signaling pathway. **c** Upregulation of miR-133b repressed the transcriptional activity based on a TGF-β/Smad-responsive luciferase reporter in the absence or presence of TGF-β (5 ng/ml). *P < 0.05. **d** Western blot analysis showing that upregulation of miR-133b decreased nuclear translocation of pSMAD3 in PCa cells in the absence or presence of TGF-β. The nuclear protein p84 was used as a nuclear protein marker. **e** Overexpressing miR-133b repressed downstream bone metastasis-related genes of the TGF-β pathway, including CTGF, PTHRP, IL-11, NEDD9, MMP13, ADAM19, THBS1, COL1A1 and VEGFA, in the absence or presence of TGF-β. Transcript levels were normalized to GAPDH expression

To investigate the underlying signaling pathways mediating the role of miR-133b in bone metastasis of PCa, luciferase reporter plasmids of multiple signaling pathways were transfected into PCa cells respectively. As shown in Fig. 3a, TGF-β/Smad-responsive luciferase reporter activity was consistently downregulated by upregulation of miR-133b in three bone metastatic PCa cells, and upregulated by silencing miR-133b in these three cells. Gene Set Enrichment Analysis (GSEA) was further analyzed based on miR-133b expression from TCGA, and the result indicated that miR-133b expression levels correlated with TGF-β signaling pathway (Fig. 3b) that has been extensively demonstrated to play a pivotal role in bone metastasis of several human cancer, including PCa^11,15,41^. Therefore, we further examined the effects of miR-133b on TGF-β signaling activity in PCa cells by exogenously overexpressing miR-133b and endogenously knocking down miR-133b via virus transduction in PCa cells (Supplementary Figure 3a). As shown in Fig. 3c and Supplementary Figure 4a, upregulating miR-133b significantly repressed, while silencing miR-133b enhanced the transcriptional activity of the TGF-β/Smad-responsive luciferase reporter in the absence or presence of ectopic TGF-β in PCa cells, and the expression levels of miR-133b in PCa cells was not affected by TGF-β treatment (Supplementary Figure 4b). Western blotting analysis revealed that upregulation of miR-133b decreased nuclear translocation of pSMAD3 in PCa cells in the absence or presence of ectopic TGF-β, whereas silencing miR-133b increased its expression (Fig. 3d and Supplementary Figure 4c). In addition, upregulation of miR-133b reduced, while silencing miR-133b increased multiple downstream bone metastasis-related genes of the TGF-β pathway in both the absence and presence of ectopic TGF-β (Fig. 3e). Importantly, the stimulatory effects of silencing miR-133b on invasion and migration abilities of PCa cells were abrogated by TGF-β signaling inhibitor SD208 (Supplementary Figure 4d). Thus, our results demonstrate that miR-133b inhibits TGF-β signaling activity in PCa cells.

### miR-133b targets TGFBI and TGFBII

**Fig. 4:**
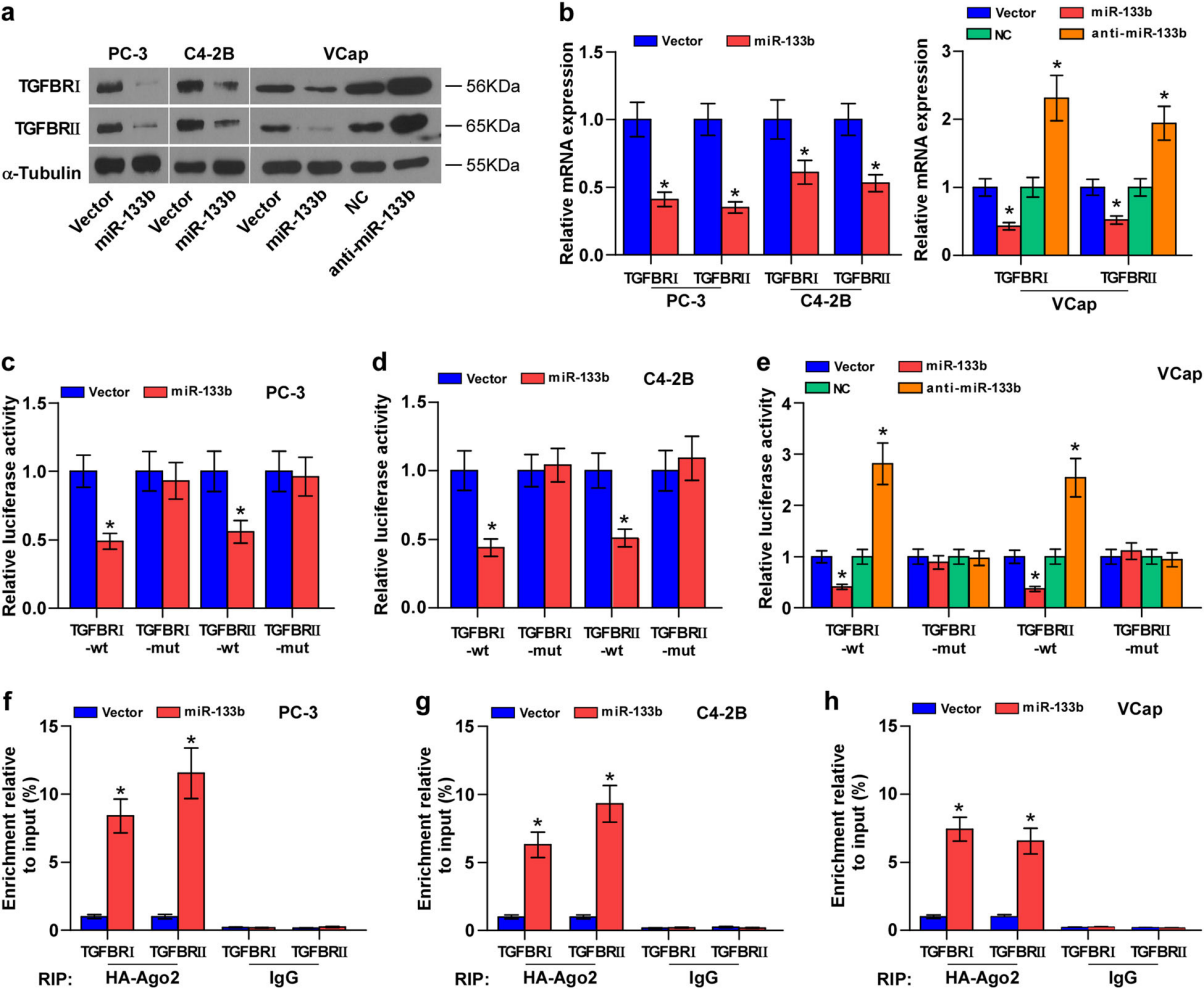
miR-133b targets TGFBRI and TGFBRII. **a** Western blotting of TGFBRI and TGFBRII expression in the indicated cells. α-Tubulin served as the loading control. **b** Real-time PCR analysis of TGFBRI and TGFBRII expression in the indicated PCa cells. Transcript levels were normalized by GAPDH expression. Error bars represent the mean ± s.d. of three independent experiments. *P < 0.05. **c**–**e** Luciferase assay of cells transfected with pmirGLO-3′-UTR reporter in the indicated PCa cells, respectively. *P < 0.05. **f**–**h** MiRNP IP assay showing the association between miR-133b and TGFBRI and TGFBRII transcripts in PCa cells. Pulldown of IgG antibody served as the negative control

By several available algorithms TargetScan and miRanda, we found that TGF-β receptor I (TGFBRI) and II (TGFBRII) may be potential target of miR-133b (Supplementary Figure 5a). RT-PCR and western blotting revealed that upregulating miR-133b reduced, while silencing miR-133b increased the mRNA and protein expression levels of TGFBRI and TGFBRII (Fig. 4a, b). Luciferase assay revealed that upregulating miR-133b decreased, while silencing miR-133b increased the reporter activity of the 3′-UTRs of TGFBRI and TGFBRII transcripts (Fig. 4c–e). Furthermore, RNA immunoprecipitation (IP) assay demonstrated a selective association of miR-133b with TGFBRI and TGFBRII transcripts (Fig. 4f–h). Furthermore, individual silencing TGFBRI or TGFBRII or both differentially attenuated the stimulatory effects of silencing miR-133b on invasion and migration abilities of PCa cells (Supplementary Figure 5b). Consequently, our results reveal that TGFBRI and TGFBRII are the direct targets of miR-133b in PCa cells.

### REST transcriptionally inhibits miR-133b expression in PCa tissues

**Fig. 5:**
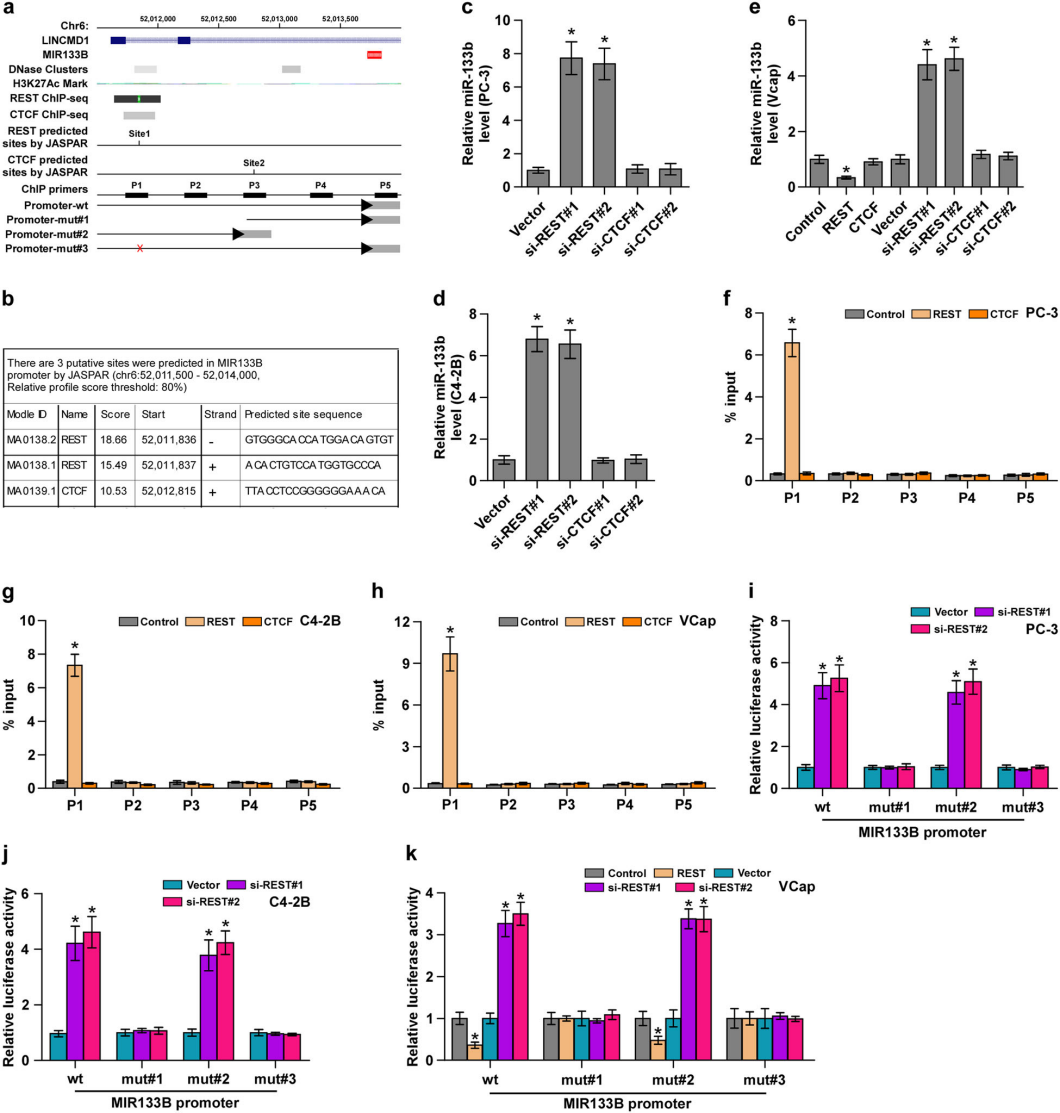
REST transcriptionally inhibits miR-133b expression in PCa cells. **a** Schematic representation of the promoter regions of MIR-133B with the putative REST and CTGF-binding sites through UCSC. **b** The putative binding sites of REST and CTGF in MIR-133B promoter by JASPAR. **c**–**e** Real-time PCR analysis of miR-133b expression in the REST or CTGF overexpressing or silencing PC-3 (**c**), C4-2B (**d**) and VCaP (**e**) cells.U6 was used as endogenous controls in RT-PCR. Each bar represents the mean values ± SD of three independent experiments. *P < 0.05. **f**–**h** Analysis of MIR-133B promoter physically associated with REST or CTGF by using chromatin immunoprecipitation (ChIP) assay in the indicated PCa cells. *P < 0.05. **i**–**k** Relative luciferase activity of the indicated promoter vectors in the indicated PCa cells. *P *<* *0.05*

To investigate the underlying mechanism responsible for miR-133b downexpression in PCa tissues, we analyzed the deletion levels in PCa data set from TCGA from a genetic perspective and found that only 6.25% of PCa tissues appeared deletion (~29 cases) (Supplementary Figure 6a). However, the expression level of miR-133b with deletion was no significant difference compared with those without deletion (Supplementary Figure 6b). This finding suggested that deletion may be not responsible for miR-133b downexpression in bone metastatic PCa tissues. Furthermore, CpG island was not found in the promoter of MIR-133B through UCSC analysis, suggesting that methylation level is not a primary mechanism regulating miR-133b expression in PCa. Numerous studies have reported that dysregulation of miRNAs frequently occurred at a transcriptional level^42^. So, we further analyzed whether some transcriptional factor may be involved in miR-133b downexpression in bone metastatic PCa tissues. The UCSC bioinformatics identified two transcriptional factors, CTGF and REST, with the potent binding ability in the promoter region of MIR-133B (Fig. 5a). Through analyzing JASPAR, several potential REST or CTGF-binding motifs were identified inside the putative promoter region of MIR-133B (Fig. 5b). RT-PCR analysis showed that upregulation of REST repressed, while silencing REST enhanced miR-133b expression levels, but CTGF had no any effect on miR-133b expression (Fig. 5c–e). A ChIP assay indicated that only REST bound to the P1-binding site in the promoter region of MIR-133B in PCa cells (Fig. 5f–h). Furthermore, upregulation of REST reduced, while silencing REST elevated the promoter luciferase activity of MIR-133B (Fig. 5i–k). However, upregulation or downregulation of REST had no effect on the luciferase activity of the MIR-133B promoter without P1-binding sites (Fig. 5i–k). These results indicated that REST transcriptionally inhibits miR-133b expression. Collectively, our results demonstrated that REST transcriptionally inhibits miR-133b expression.

### Clinical relation of miR-133b with TGFBRI, TGFBRII and REST expression, and TGF-β signaling activity in human PCa tissues

**Fig. 6:**
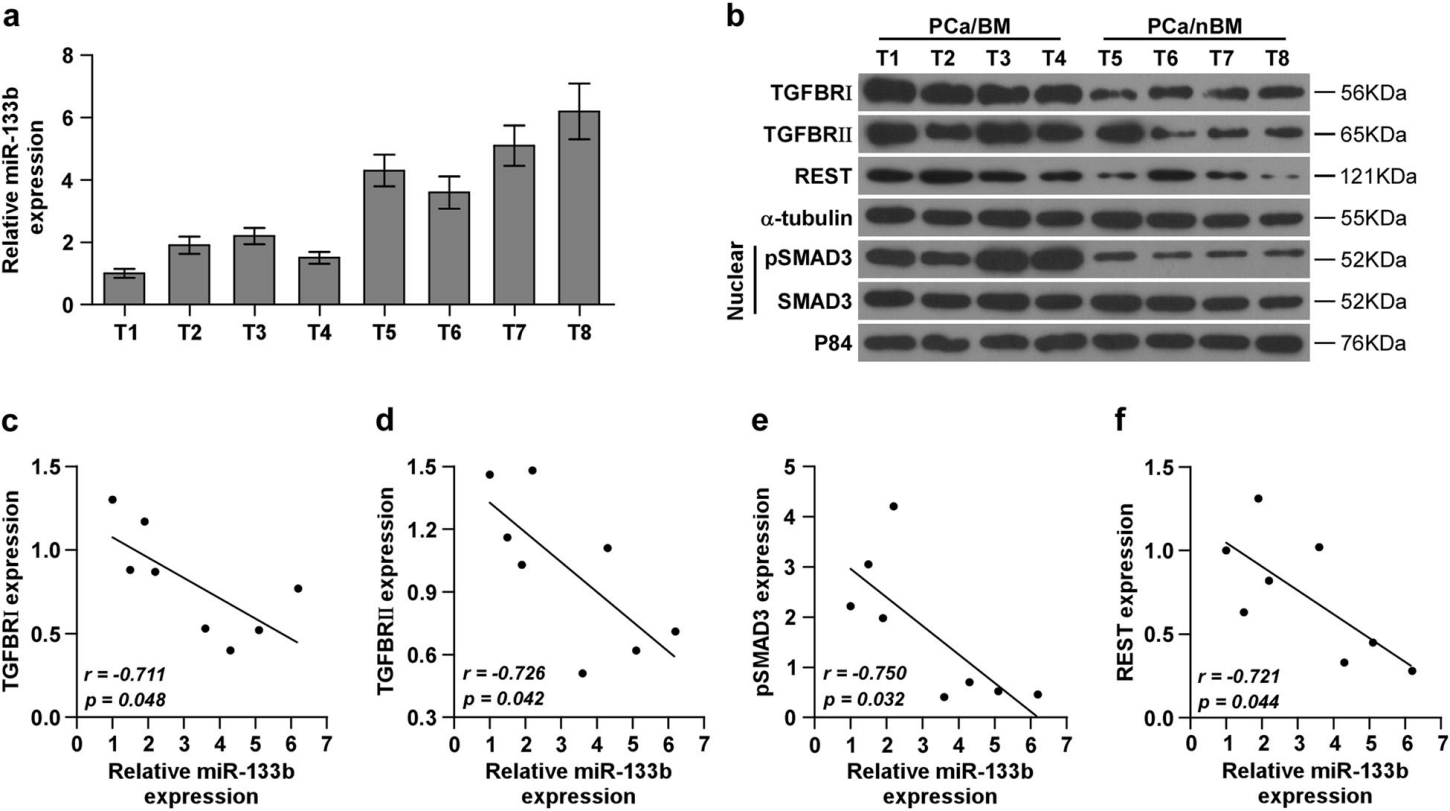
Clinical relevance of miR-133b with TGFBRI, TGFBRII and REST expression, and TGF-β signaling activity in PCa tissues. **a**, **b** Analysis of miR-133b expression with TGFBRI, TGFBRII and REST, and nuclear pSMAD3 expression in 4 bone metastatic PCa tissues (T1–4) and 4 non-bone metastatic PCa tissues (T5–8). U6 was used as the control for RNA loading. miR-133b expression levels were normalized to that miR-133b expression of sample one. Each bar represents the mean ± SD of three independent experiments. *P < 0.05. Loading controls were α-tubulin and p84 for the cytoplasmic and nuclear fractions respectively. **c**–**f** Correlation between miR-133b levels and TGFBRI, TGFBRII and REST, and nuclear pSMAD3 expression in PCa tissues.The expression levels of TGFBRI, TGFBRII and REST, and nuclear pSMAD3 expression were quantified by densitometry using Image J, and normalized to the levels of α-tubulin and p84, respectively. The sample 1 was used as a standard. The relative expressions of miR-133b and these proteins were used to perform the correlation analysis

To determine the clinical correlation of miR-133b with TGFBRI, TGFBRII, REST expression, and TGF-β signaling activity in clinical PCa tissues, the miR-133b expression and protein levels of TGFBRI, TGFBRII, REST and nuclear SMAD2/3 expression were examined in four bone metastatic and four non-bone metastatic PCa tissues. As shown in Fig. 6a, b, protein expression of TGFBRI, TGFBRII, REST and nuclear pSMAD3 in bone metastatic PCa tissues (T1–4) was upregulated compared with that in non-bone metastatic PCa tissues (T5–8). Conversely, miR-133b displayed an opposite pattern. Pearson analysis revealed that miR-133b expression inversely correlated with TGFBRI, TGFBRII and REST expression, and nuclear pSMAD3 expression (Fig. 6c–f.). Taken together, our results indicate that transcriptional repression of miR-133b by REST in bone metastatic PCa tissues activates TGF-β signaling by upregulating TGFBRI and TGFBRII expression, finally promoting the development of PCa bone metastasis.

### Overexpression of miR-133b represses bone metastasis of PCa cells in vivo

**Fig. 7:**
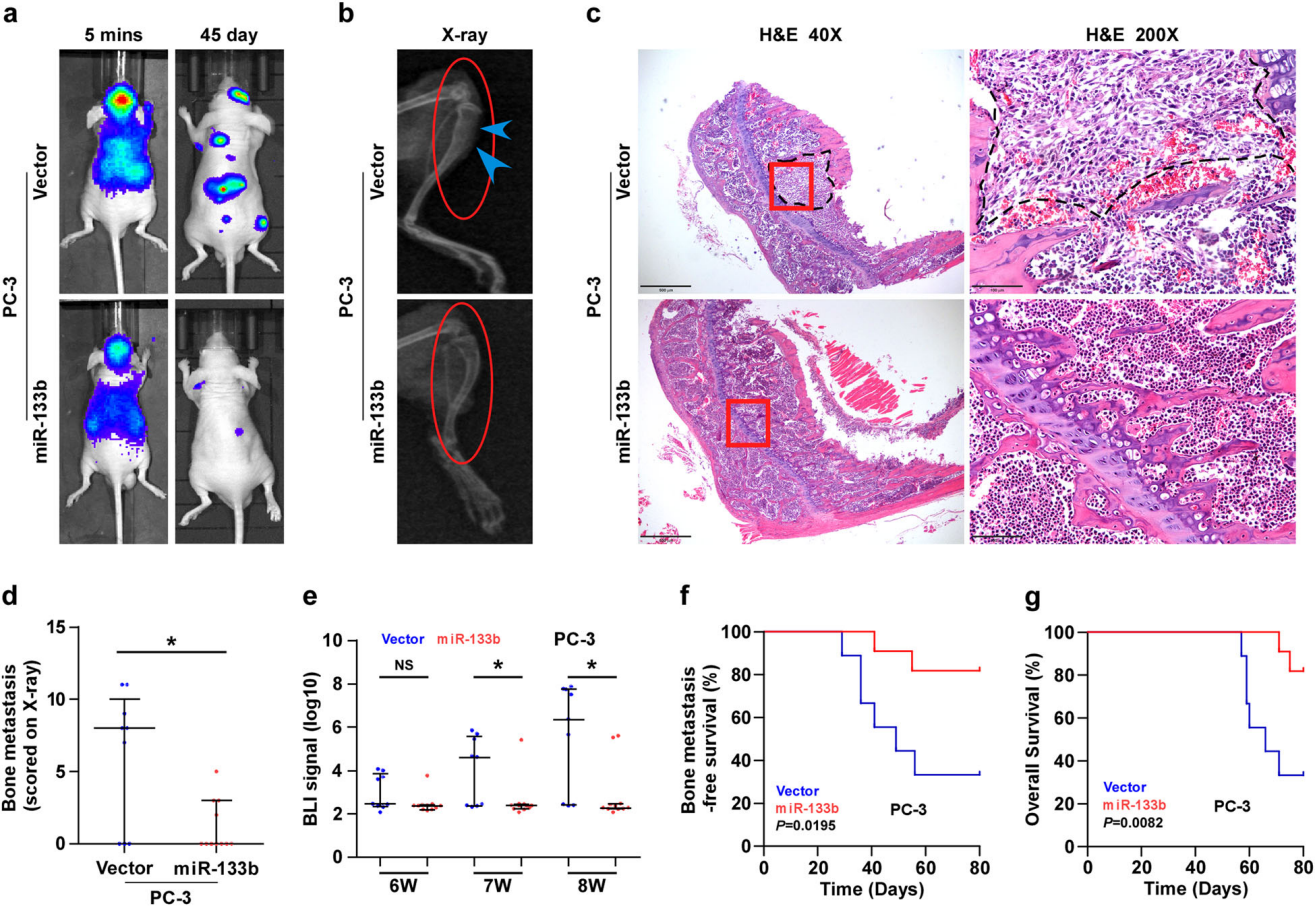
Upregulating miR-133b represses bone metastasis of PC-3 cells in vivo. **a** Representative BLIs signal of bone metastasis of a mouse from the indicated groups of mice at 5 mins and 45 day, respectively. **b** Representative radiographic images of bone metastases in the indicated mice (arrows indicate osteolytic lesions). **c** Representative H&E-stained sections of tibias from the indicated mouse. **d** The sum of bone metastasis scores for each mouse in tumor-bearing mice inoculated with vector (*n* = 9) or miR-133b (*n* = 11) cells. **e** Quantification of the BLI signaling in the vector and miR-133b-overexpression groups at 6, 7 and 8 weeks, respectively. *P *<* 0.05. **f** Kaplan–Meier analysis of mouse bone metastasis-free survival in the vector and miR-133b-overexpression groups. The mean survival times: vector = 49 days, and miR-133b > 80 days. **g** Kaplan–Meyer analysis of mouse survival in the vector and miR-133b-overexpression groups. The mean survival times: vector = 66 days, and miR-133b > 80 days. NS no significance

Our results above indicated that miR-133b was downregulated in bone metastasis PCa tissues, which positively correlated with poor bone metastasis-free survival in PCa patients. Therefore, we further investigated the effect of miR-133b on the bone metastasis of PCa in vivo. A mouse model of bone metastasis was used, in which the luciferase-labeled vector or miR-133b-overexpressing PC-3 cells were inoculated, respectively, into the left cardiac ventricle of male nude mice to monitor the progression of bone metastasis by bioluminescence imaging (BLI). As shown in Fig. 7a, b, ectopic expression of miR-133b dramatically suppressed bone metastasis ability compared with the control group by X-ray and BLI. H&E staining of the tumor sections from the indicated mice tibias showed that upregulating miR-133b decreased the tumor burden in bone (Fig. 7c). Furthermore, mice injected with miR-133b-overexpressing cells exhibited fewer bone metastatic sites and smaller osteolytic area of metastatic tumors, as well as longer survival and bone metastasis-free survival compared to the control group (Fig. 7d–g). Furthermore, upregulating miR-133b repressed, while silencing miR-133b increased invasion and migration abilities of PCa cells (Supplementary Figure 7a–d). Consistently, upregulating miR-133b reduced, while silencing miR-133b enhanced several migration and invasion markers, including MMP9, MMP13 and TIMP2 (Supplementary Figure 7e and f). Consequently, our results demonstrate that upregulating miR-133b represses the bone metastasis of PCa in vivo.

### REST promotes bone metastasis via inhibiting miR-133b in PCa cells

**Fig. 8:**
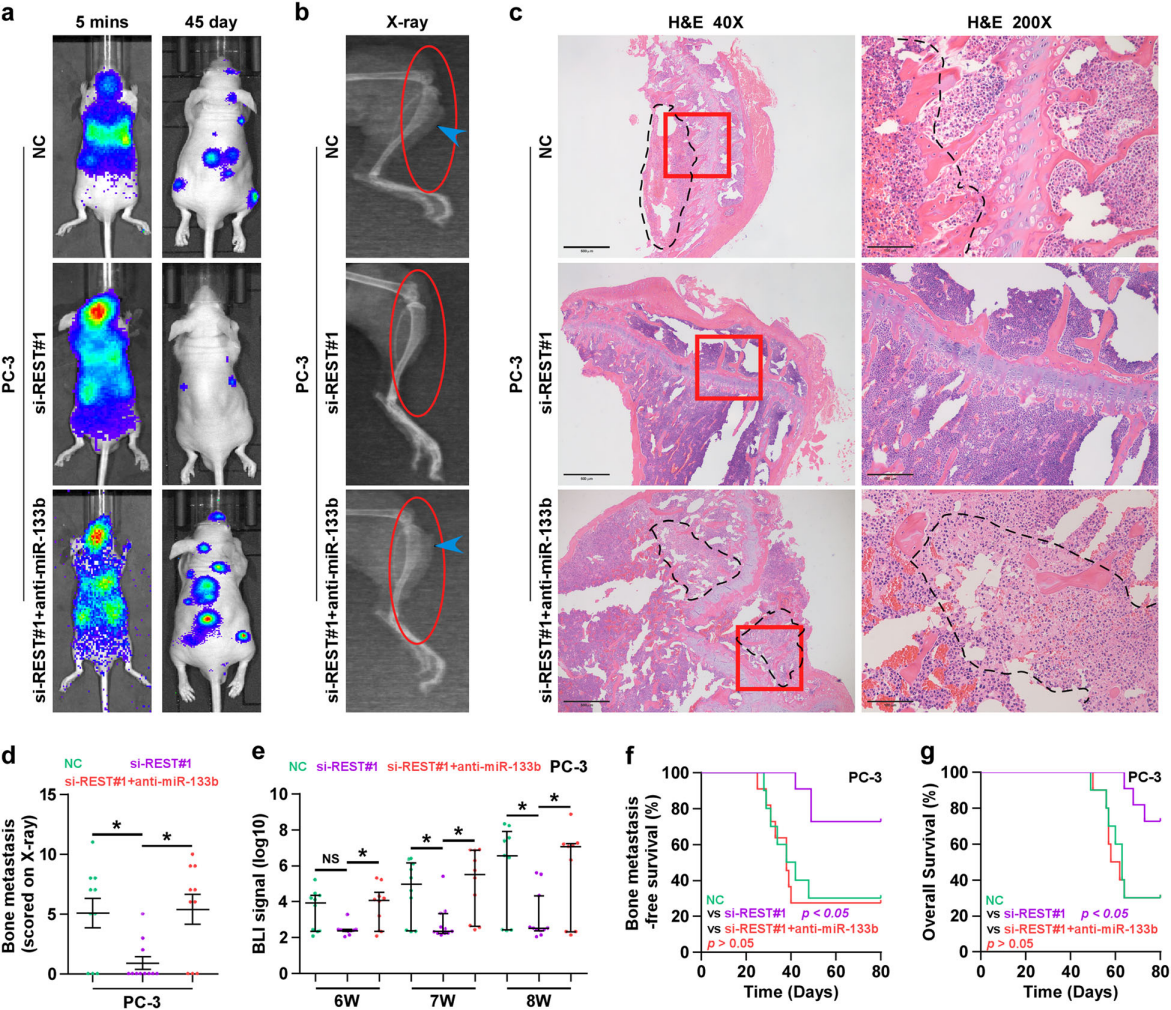
REST promotes bone metastasis via inhibiting miR-133b in PCa cells in vivo. **a** Representative BLIs signal of bone metastasis of a mouse from the indicated groups of mice at 5 mins and 45 days, respectively. **b** Representative radiographic images of bone metastases in the indicated mice (arrows indicate osteolytic lesions). **c** Representative H&E-stained sections of tibias from the indicated mouse. **d** The sum of bone metastasis scores for each mouse in tumor-bearing mice inoculated with vector (*n* = 10), siREST (*n* = 11) or siREST-anti-miR-133b (*n* = 10) cells. **e** Quantification of the BLI signaling in the vector and miR-133b-overexpression groups at 6, 7 and 8 weeks, respectively. *P *<* 0.05. **f** Kaplan–Meier analysis of mouse bone metastasis-free survival in the vector, siREST and siREST-anti-miR-133b groups. The mean survival times: NC = 40 days, si-REST#1 > 80 days and si-REST#1 + anti-miR-133b = 38 days. **g** Kaplan–Meyer analysis of mouse survival in the vector, siREST and siREST-anti-miR-133b groups. The mean survival times: NC = 63 days, si-REST#1 > 80 days and si-REST#1 + anti-miR-133b = 60 days. NS no significance

We further evaluated whether miR-133b mediated the function role of REST in bone metastasis of PCa. We first examined the effect of REST on bone metastasis of PCa in vivo. As shown in Fig. 8a–g, we found that silencing REST repressed bone metastasis ability of PC-3 cells, including decreasing bone metastatic score and osteolytic area of tumors, and prolonging bone metastasis-free survival compared to the control group by bioluminescence imaging (BLI), X-ray, H&E staining and survival analysis. Next, to investigate the role of REST/miR-133b axis in bone metastasis of PCa, we further downregulating miR-133b in REST-silenced PC-3 cells, and we found that silencing miR-133b enhanced bone metastasis ability in vivo in REST-silenced PCa cells (Fig. 8a–g). Consistently, silencing miR-133b enhanced the TGF-β signaling activity, invasion and migration abilities in REST-silencing PCa cells (Supplemental Fig. 8a–d). These findings indicated that REST activates TGF-β signaling, and promotes bone metastasis via inhibiting miR-133b in PCa cells.

## Discussion

In the present study, we reported that miR-133b was downregulated in PCa tissues, particularly in bone metastatic PCa tissues. Importantly, low expression of miR-133b correlated with advanced clinicopathological characteristics and poor bone metastasis-free survival in PCa patients. Our results further revealed that miR-133b inhibited invasion and bone metastasis of PCa cells via targeting TGFBRI and TGFBRII, leading to the inactivation of TGF-β signaling. These results determine the tumor-suppressive role of miR-133b in bone metastasis of PCa.

Constitutive activation of TGF-β signaling has been reported in bone metastases of various types of cancer^11,14,15^, to which several regulatory mechanisms contributed, such as increase of autocrine TGF-β, mutation of core components of TGF-β signaling^43–45^. Recent studies have demonstrated that aberrant of miRNAs is another important mechanism responsible for activation of TGF-β signaling, giving rise to tumor cell metastasis in different types of cancer. The study by Bonci has reported that simultaneous loss of miR-15/miR-16 in PCa cells significantly activated TGF-β signaling, which further promoted invasion, distant bone marrow colonization and development of osteolytic tumor^46^. In this study, we found that downxpression of miR-133b repressed the activity of TGF-β signaling via targeting TGFBRI and TGFBRII in PCa cells, which further inhibited bone metastasis of PCa cells. Taken together, our findings unravel a novel mechanism of TGF-β signaling-mediated bone metastasis of PCa.

As one of the originally identified muscle-specific microRNAs, the roles of miR-133b in the development and remodeling of heart and skeletal muscle have been well documented^47,48^. Furthermore, numerous studies have shown that miR-133b comes into playing important pathological role in human cancer^49^. In gastric cancer, bladder cancer, renal cell carcinoma, squamous cell carcinoma of esophagus and tongue, miR-133b has been reported to be downregulated and low expression of miR-133b promoted the progression and metastasis via vary mechanism^50–54^. However, high expression of miR-133b has been demonstrated in cervical carcinoma and glioblastoma,^55,56^. There studies indicated that the pro- and anti-tumor roles of miR-133b are environment and tumor-type dependent. Interestingly, a study by Li et al. has shown that overexpression of miR-133b in LNCaP cells boosted cell proliferation and cell-cycle progression, but inhibited apoptosis; in contrast, upregulation of miR-133b yielded an opposite role in PC-3 cells, indicating that miR-133b might enhance tumor-promoting properties in less aggressive LNCaP cells, whereas this miR may act as a tumor suppressor in more aggressive PC-3 cells^57^. Furthermore, several lines of evidence have shown that miR-133b was downregulated in PCa tissues and acted as a tumor-suppressive miRNA in PCa^58–61^. Importantly, Coarfa and colleagues reported that miR-133b was dramatically downexpressed in metastatic PCa tissues^62^. These studies support the ideal that miR-133b is downregulated in PCa tissues, which was involved in the metastatic phenotype of PCa. However, the clinical significance and biological roles of miR-133b in bone metastasis of PCa remain unclear. In this study, our results showed that miR-133b was downregulated in PCa tissues and further decreased in bone metastatic PCa tissues, which was associated with poor clinicopathological characteristics and bone metastasis-free survival in PCa patients. In addition, upregulating miR-133b dramatically inhibited invasion and migration in vitro, and bone metastasis in vivo in PCa cells by repressing activity of TGF-β signaling via targeting TGFBRI and TGFBRII in PCa cells. Therefore, our results indicate that miR-133b plays an inhibitory role in bone metastasis of PCa.

As discussed above, the downexpression of miR-133b has been widely reported in PCa. However, the underlying mechanism responsible for miR-133b downexpression in cancers was not clarified. With this question, we first analyzed the deletion levels of miR-133b in the PCa data set from TCGA and found that 6.25% of 496 PCa tissues occurred deletions, but the miR-133b expression levels in PCa tissues with deletions was no significant difference compared with those without gains. This finding suggested that deletion was not responsible for miR-133b downexpression in PCa tissues. Numerous studies have reported that dysregulation of miRNAs frequently occurred at a transcriptional level^42^. In this study, our results demonstrated that REST transcriptionally inhibited miR-133b expression, which resulted in miR-133b downexpression in PCa tissues. These results indicated that overexpression of REST contributed to miR-133b downexpression in PCa tissues. Indeed, REST has been extensively demonstrated to be a transcriptional repressor involved in the control of neuroendocrine gene expression^63,64^, and overexpression of REST has previously been reported in various types of cancer^21,22,65^. Importantly, a study by Epping has reported that the TSPYL2/REST complex activated TGF-β signaling in breast cancer^66^. However, how REST promotes TGF-β signaling remains unknown. In this study, our results found that REST transcriptionally inhibited miR-133b expression, and miR-133b repressed activity of TGF-β signaling via targeting TGFBRI and TGFBRII, indicating that REST activates TGF-β signaling via transcriptional repression of miR-133b. Therefore, our results in combination with other reveal that REST/miR-133b axis promotes bone metastasis via constitutively activating TGF-β signaling in PCa.

Several lines of evidence reported that miR-133b was identified in the serum of cancer patients, suggesting that miR-133b may serve as a potential diagnostic and prognostic marker in human cancers. Zhang et al. reported that expression levels of miR-133b were significantly decreased in the serum of osteosarcoma patients compared with the healthy controls^67^. Moreover, high expression level of miR-133b expression was identified in the serum of breast cancer patients^68^. Interestingly, miRNA profiling of prostate secretion samples (PSS) from 23 PCa and 25 benign prostate hyperplasia (BPH) patients revealed that miR-133b was significantly downregulated in PSS of PCa patients. Importantly, miR-133b has much power than PSA to discriminate PCa from BPH patients^59^, suggesting that miR-133b expression levels in PSS could be used as diagnostics markers for PCa. In this study, our results showed that miR-133b expression inversely correlated with bone metastasis-free survival in PCa patients, suggesting that miR-133b may be used as a potential bone metastasis diagnostic marker in PCa cancers. However, whether miR-133b expression in the serum or prostate secretion samples of PCa patients may be used as a minimal invasive diagnostic marker for the bone metastasis of PCa needs to be further validated in a larger series of studies.

In summary, our results indicate that miR-133b inhibits bone metastasis of PCa by targeting TGFBRI and TGFBRII, leading to inactivation of TGF-β signaling pathway, suggesting that administration of miR-133b may serve as a therapeutic strategy in the treatment of PCa bone metastasis. Better understanding of the underlying mechanism of miR-133b in the inactivation of TGF-β signaling will facilitate the development of novel anti-metastatic therapeutic methods against PCa.

## Materials and Methods

### Cell lines and cell culture

The human PCa cell lines 22RV1, PC-3, VCaP, DU145, LNCaP and normal prostate epithelial cells RWPE-1 were obtained from Shanghai Chinese Academy of Sciences cell bank (China). RWPE-1 cells were grown in defined keratinocyte-SFM (1×) (Invitrogen). PC-3, LNCaP and 22Rv1 cells were cultured in RPMI-1640 medium (Life Technologies, Carlsbad, CA, US) supplemented with penicillin G (100 U/ml), streptomycin (100 mg/ml) and 10% fetal bovine serum (FBS, Life Technologies). DU145 and VCaP cells were grown in Dulbecco’s modified Eagle’s medium (Invitrogen) supplemented with 10% FBS. The C4-2B cell line was purchased from the MD Anderson Cancer Center and maintained in T-medium (Invitrogen) supplemented with 10 % FBS. All cell lines were grown under a humidified atmosphere of 5% CO2 at 37 °C.

### Plasmids, transfection and generation of stable cell lines

The human miR-133b gene was PCR-amplified from genomic DNA and cloned into a pMSCV-puro retroviral vector (Clontech, Japan). The (CAGAC) 12/pGL3 TGF-β/Smad-responsive luciferase reporter plasmid and control plasmids (Clontech, Japan) were used to quantitatively assess the transcriptional activity of TGF-β signaling components. The 3′-UTR of TGFBRI and TGFBRII were PCR-amplified from genomic DNA and cloned into pmirGLO vectors (Promega, USA), and the list of primers used in cloning reactions is shown in Supplementary Table 4. AntagomiR-133b, small interfering RNA (siRNA) for TGFBRI and TGFBRII knockdown and corresponding control siRNAs were synthesized and purified by RiboBio. Human REST cDNA was purchased form (Vigene Biosciences, Shandong, China). Transfection of miRNA, siRNAs, and plasmids was performed using Lipofectamine 3000 (Life Technologies, USA) according to the manufacturer’s instructions.

### RNA extraction, reverse transcription, and real-time RT-PCR

Total RNA from tissues or cells was extracted using the RNA Isolation Kit (Qiagen, USA) according to the manufacturer’s instructions. Messenger RNA (mRNA) and miRNA were reverse transcribed from total mRNA using the RevertAid First Strand cDNA Synthesis Kit (Thermo Fisher, USA) according to the manufacturer’s protocol. Complementary DNA (cDNA) was amplified and quantified on the CFX96 system (BIO-RAD, USA) using iQ SYBR Green (BIO-RAD, USA). The primers are provided in Supplementary Table 5. Primers for U6 and miR-133b were synthesized and purified by RiboBio (Guangzhou, China). U6 or glyceraldehyde-3-phosphate dehydrogenase (GAPDH) was used as the endogenous controls. Relative fold expressions were calculated with the comparative threshold cycle (2^-ΔΔCt^) method.

### Patients and tumor tissues

A total of 182 individual and 20 paired PCa tissues, and 48 benign prostate lesions tissues were obtained during surgery or needle biopsy at The Clinical Biobank of Collaborative Innovation Center for Medical Molecular Diagnostics of Guangdong Province, The Affiliated Jiangmen Hospital of Sun Yat-sen University (Guangdong, China), and the Second Affiliated Hospital of Guangzhou Medical University (Guangdong, China) between January 2008 and December 2016. Patients were diagnosed based on clinical and pathological evidence, and the specimens were immediately snap-frozen and stored in liquid nitrogen tanks. For the use of these clinical materials for research purposes, prior patient’ consents and approval from the Institutional Research Ethics Committee were obtained. The clinicopathological features of the patients are summarized in Supplementary Table 6-8. The median of miR-133b expression in PCa tissues was used to stratify high and low expression of miR-133b.

### miRNA immunoprecipitation

Cells were co-transfected with HA-Ago2, followed by HA-Ago2 immunoprecipitation using anti-HA-antibody. Real-time PCR analysis of the IP material was performed to test the association of the mRNA of TGFBRI and TGFBRII with the RISC complex. The specific processes were performed as previously described^69^.

### Western blot

Western blot was performed according to a standard method, as previously described^70^. Antibodies against TGFBRI, anti–TGFBRII and p84 were purchased from Abcam, anti–pSMAD3 and anti–SMAD3 from Cell Signaling Technology. Nuclear extracts were prepared using the Nuclear Extraction Kit (Active Motif), according to the manufacturer’s instructions. As a loading control, membranes were stripped and reprobed with an anti-α-tubulin antibody (Sigma-Aldrich, USA).

### Luciferase reporter assay

Cells (4 × 10^4^) were seeded in triplicate in 24-well plates and cultured for 24 h and performed as previously described^71^. Luciferase and Renilla signals were measured 36 h after transfection using a Dual Luciferase Reporter Assay Kit (Promega).

### Chromatin immunoprecipitation (ChIP)

Cells were cultured as described above. Cross-linking was performed with formaldehyde (Merck) at a final concentration of 1 % and terminated after 5 min by the addition of glycine at a final concentration of 0.125 M. Cells were collected with SDS buffer, pelleted by centrifugation, and resuspended in IP buffer. Chromatin was sheered by sonication (HTU SONI 130; Heinemann) to generate DNA fragments with an average size of 500 bp. Preclearing and incubation with anti-Flag (F1804, Sigma) antibodies or IgG control (M-7023, Sigma) for 16 h was performed. Immunoprecipitated DNA was decrosslinked, digested with proteinase K and purified. Immunoprecipitated DNA was analyzed by qPCR and the primers of MIR133B promoter was presented in Supplementary Table 4. Enrichment was expressed as the percentage of the input for each condition.

### Invasion and migration assays

The invasion and migration assays were performed as described previously^72^. The cell count was performed under a microscope (x100).

### Animal study

All mouse experiments were approved by The Institutional Animal Care and Use Committee of Sun Yat-sen University and the approval-No. was L102012016110D. For the bone metastasis study, BALB/c-nu mice (5–6 weeks old) were anaesthetized and inoculated into the left cardiac ventricle with 1 × 10^5^ PC-3 cells in 100 μl of PBS. Osteolytic lesions were identified on radiographs as radiolucent lesions in the bone. The area of the osteolytic lesions was measured using the Metamorph image analysis system and software (Universal Imaging Corporation), and the total extent of bone destruction per animal was expressed in square millimeters. Each bone metastasis was scored based on the following criteria: 0, no metastasis; 1, bone lesion covering < 1/4 of the bone width; 2, bone lesion involving 1/4–1/2 of the bone width; 3, bone lesion across 1/2–3/4 of the bone width; and 4, bone lesion > 3/4 of the bone width. The bone metastasis score for each mouse was the sum of the scores of all bone lesions from four limbs.

### Statistical analysis

All values are presented as the mean ± SD. Significant differences were determined using the GraphPad 5.0 software (USA). Student’s *t* test was used to determine statistical differences between two groups. The *Χ*^2^ test was used to analyze the relationship between miR-133b expression and clinicopathological characteristics. Survival curves were plotted using the Kaplan–Meier method and compared by log-rank test. *P* < 0.05 was considered statistical significant. All experiments were repeated three times.

## Electronic supplementary material

Supplementary Table 1

Supplementary Table 2

Supplementary Table 3

Supplemental Table 4

Supplemental Table 5

Supplementary Table 6

Supplementary Table 7

Supplementary Table 8

Supplemental Figure legends

Supplemental Figure 1

Supplemental Figure 2

Supplemental Figure 3

Supplemental Figure 4

Supplemental Figure 5

Supplemental Figure 6

Supplemental Figure 7

Supplemental Figure 8

## References

[1] Torre LA (2015). Global cancer statistics, 2012. CA Cancer J. Clin..

[2] Chen W (2016). Cancer statistics in China, 2015. CA Cancer J. Clin..

[3] Body JJ, Casimiro S, Costa L (2015). Targeting bone metastases in prostate cancer: improving clinical outcome. Nat. Rev. Urol..

[4] Weinfurt KP (2005). The significance of skeletal-related events for the health-related quality of life of patients with metastatic prostate cancer. Ann. Oncol..

[5] Langley RR, Fidler IJ (2011). The seed and soil hypothesis revisited–the role of tumor-stroma interactions in metastasis to different organs. Int. J. Cancer.

[6] Saad F (2007). Pathologic fractures correlate with reduced survival in patients with malignant bone disease. Cancer.

[7] Mohammad KS (2009). Pharmacologic inhibition of the TGF-beta type I receptor kinase has anabolic and anti-catabolic effects on bone. PLoS ONE.

[8] Shi Y, Massague J (2003). Mechanisms of TGF-beta signaling from cell membrane to the nucleus. Cell.

[9] Huse M (2001). The TGF beta receptor activation process: an inhibitor- to substrate-binding switch. Mol. Cell.

[10] Pickup M, Novitskiy S, Moses HL (2013). The roles of TGFbeta in the tumour microenvironment. Nat. Rev. Cancer.

[11] Kang Y (2005). Breast cancer bone metastasis mediated by the Smad tumor suppressor pathway. Proc. Natl Acad. Sci. USA.

[12] Sethi N, Dai X, Winter CG, Kang Y (2011). Tumor-derived JAGGED1 promotes osteolytic bone metastasis of breast cancer by engaging notch signaling in bone cells. Cancer Cell.

[13] Kang Y (2003). A multigenic program mediating breast cancer metastasis to bone. Cancer Cell.

[14] Yin JJ (1999). TGF-beta signaling blockade inhibits PTHrP secretion by breast cancer cells and bone metastases development. J. Clin. Invest..

[15] Javelaud D (2007). Stable overexpression of Smad7 in human melanoma cells impairs bone metastasis. Cancer Res..

[16] Fournier PG (2015). The TGF-beta signaling regulator PMEPA1 suppresses prostate cancer metastases to bone. Cancer Cell.

[17] Hu Z (2012). Systemic delivery of oncolytic adenoviruses targeting transforming growth factor-beta inhibits established bone metastasis in a prostate cancer mouse model. Hum. Gene Ther..

[18] Wan X (2012). Effect of transforming growth factor beta (TGF-beta) receptor I kinase inhibitor on prostate cancer bone growth. Bone.

[19] Mori N, Schoenherr C, Vandenbergh DJ, Anderson DJ (1992). A common silencer element in the SCG10 and type II Na + channel genes binds a factor present in nonneuronal cells but not in neuronal cells. Neuron.

[20] Chong JA (1995). REST: a mammalian silencer protein that restricts sodium channel gene expression to neurons. Cell.

[21] Blom T (2006). Molecular genetic analysis of the REST/NRSF gene in nervous system tumors. Acta Neuropathol..

[22] Lawinger P (2000). The neuronal repressor REST/NRSF is an essential regulator in medulloblastoma cells. Nat. Med..

[23] Fuller GN (2005). Many human medulloblastoma tumors overexpress repressor element-1 silencing transcription (REST)/neuron-restrictive silencer factor, which can be functionally countered by REST-VP16. Mol. Cancer Ther..

[24] Kreisler A (2010). Regulation of the NRSF/REST gene by methylation and CREB affects the cellular phenotype of small-cell lung cancer. Oncogene.

[25] Terry S, Beltran H (2014). The many faces of neuroendocrine differentiation in prostate cancer progression. Front. Oncol..

[26] Tawadros T (2005). IB1/JIP-1 controls JNK activation and increased during prostatic LNCaP cells neuroendocrine differentiation. Cell Signal..

[27] Bartel DP (2009). MicroRNAs: target recognition and regulatory functions. Cell.

[28] Velu VK, Ramesh R, Srinivasan AR (2012). Circulating microRNAs as biomarkers in health and disease. J. Clin. Diagn. Res..

[29] Ren D (2017). Maintenance of cancer stemness by miR-196b-5p contributes to chemoresistance of colorectal cancer cells via activating STAT3 signaling pathway. Oncotarget.

[30] Zhang X (2015). Upregulation of miR-572 transcriptionally suppresses SOCS1 and p21 and contributes to human ovarian cancer progression. Oncotarget.

[31] Hu G (2017). MicroRNA-145 attenuates TNF-alpha-driven cartilage matrix degradation in osteoarthritis via direct suppression of MKK4. Cell Death Dis..

[32] Feliciano A (2017). miR-99a reveals two novel oncogenic proteins E2F2 and EMR2 and represses stemness in lung cancer. Cell Death Dis..

[33] Yang RM (2017). miR-3656 expression enhances the chemosensitivity of pancreatic cancer to gemcitabine through modulation of the RHOF/EMT axis. Cell Death Dis..

[34] Ji Q (2017). miR-216a inhibits osteosarcoma cell proliferation, invasion and metastasis by targeting CDK14. Cell Death Dis..

[35] Ren D (2017). Oncogenic miR-210-3p promotes prostate cancer cell EMT and bone metastasis via NF-kappaB signaling pathway. Mol. Cancer.

[36] Colden M (2017). MicroRNA-466 inhibits tumor growth and bone metastasis in prostate cancer by direct regulation of osteogenic transcription factor RUNX2. Cell Death Dis..

[37] Siu MK (2015). Transforming growth factor-beta promotes prostate bone metastasis through induction of microRNA-96 and activation of the mTOR pathway. Oncogene.

[38] Ren D (2013). Wild-type p53 suppresses the epithelial-mesenchymal transition and stemness in PC-3 prostate cancer cells by modulating miR145. Int. J. Oncol..

[39] Ren D (2014). Double-negative feedback loop between ZEB2 and miR-145 regulates epithelial-mesenchymal transition and stem cell properties in prostate cancer cells. Cell Tissue Res..

[40] Guo W (2013). HEF1 promotes epithelial–mesenchymal transition and bone invasion in prostate cancer under the regulation of microRNA-145. J. Cell. Biochem..

[41] Dai Y (2017). The TGF-beta signalling negative regulator PICK1 represses prostate cancer metastasis to bone. Br. J. Cancer.

[42] Chang YS (2015). EGF receptor promotes prostate cancer bone metastasis by downregulating miR-1 and activating TWIST1. Cancer Res..

[43] Macias MJ, Martin-Malpartida P, Massague J (2015). Structural determinants of Smad function in TGF-beta signaling. Trends Biochem. Sci..

[44] Bhola NE (2013). TGF-beta inhibition enhances chemotherapy action against triple-negative breast cancer. J. Clin. Invest..

[45] Scheel C (2011). Paracrine and autocrine signals induce and maintain mesenchymal and stem cell states in the breast. Cell.

[46] Bonci D (2016). A microRNA code for prostate cancer metastasis. Oncogene.

[47] Nohata N, Hanazawa T, Enokida H, Seki N (2012). microRNA-1/133a and microRNA-206/133b clusters: dysregulation and functional roles in human cancers. Oncotarget.

[48] Mitchelson KR, Qin WY (2015). Roles of the canonical myomiRs miR-1, -133 and -206 in cell development and disease. World J. Biol. Chem..

[49] Li D (2017). miR-133b, a particular member of myomiRs, coming into playing its unique pathological role in human cancer. Oncotarget.

[50] Liu Y (2015). Identification of miRNomes in human stomach and gastric carcinoma reveals miR-133b/a-3p as therapeutic target for gastric cancer. Cancer Lett..

[51] Pignot G (2013). microRNA expression profile in a large series of bladder tumors: identification of a 3-miRNA signature associated with aggressiveness of muscle-invasive bladder cancer. Int. J. Cancer.

[52] Hidaka H (2012). Tumor suppressive microRNA-1285 regulates novel molecular targets: aberrant expression and functional significance in renal cell carcinoma. Oncotarget.

[53] Kano M (2010). miR-145, miR-133a and miR-133b: tumor-suppressive miRNAs target FSCN1 in esophageal squamous cell carcinoma. Int. J. Cancer.

[54] Wong TS (2008). Mature miR-184 as potential oncogenic microRNA of squamous cell carcinoma of tongue. Clin. Cancer Res..

[55] Qin W (2012). MicroRNA-133b is a key promoter of cervical carcinoma development through the activation of the ERK and AKT1 pathways. Oncogene.

[56] Xu G, Li JY (2016). Differential expression of PDGFRB and EGFR in microvascular proliferation in glioblastoma. Tumour Biol..

[57] Li X (2014). Identification of miR-133b and RB1CC1 as independent predictors for biochemical recurrence and potential therapeutic targets for prostate cancer. Clin. Cancer Res..

[58] Pashaei E, Pashaei E, Ahmady M, Ozen M, Aydin N (2017). Meta-analysis of miRNA expression profiles for prostate cancer recurrence following radical prostatectomy. PLoS ONE.

[59] Guzel E (2015). Identification of microRNAs differentially expressed in prostatic secretions of patients with prostate cancer. Int. J. Cancer.

[60] Karatas OF (2014). miR-1 and miR-133b are differentially expressed in patients with recurrent prostate cancer. PLoS ONE.

[61] Patron JP (2012). MiR-133b targets antiapoptotic genes and enhances death receptor-induced apoptosis. PLoS ONE.

[62] Coarfa C (2016). Comprehensive proteomic profiling identifies the androgen receptor axis and other signaling pathways as targets of microRNAs suppressed in metastatic prostate cancer. Oncogene.

[63] Lim JS (2017). Intratumoural heterogeneity generated by Notch signalling promotes small-cell lung cancer. Nature.

[64] Abderrahmani A (2001). The transcriptional repressor REST determines the cell-specific expression of the human MAPK8IP1 gene encoding IB1 (JIP-1). Mol. Cell Biol..

[65] Guardavaccaro D (2008). Control of chromosome stability by the beta-TrCP-REST-Mad2 axis. Nature.

[66] Epping MT (2015). TSPYL2 is an essential component of the REST/NRSF transcriptional complex for TGFbeta signaling activation. Cell Death Differ..

[67] Zhang C, Yao C, Li H, Wang G, He X (2014). Serum levels of microRNA-133b and microRNA-206 expression predict prognosis in patients with osteosarcoma. Int. J. Clin. Exp. Pathol..

[68] Chan M (2013). Identification of circulating microRNA signatures for breast cancer detection. Clin. Cancer Res..

[69] Li, X. et al. miR150 inhibits proliferation and tumorigenicity via retarding G1/S phase transition in nasopharyngeal carcinoma. *Int. J. Oncol.* 1097–1108 (2017) 10.3892/ijo.2017.3909. 10.3892/ijo.2017.3909 PMC5363880 28350089

[70] Wang M (2016). N-cadherin promotes epithelial-mesenchymal transition and cancer stem cell-like traits via ErbB signaling in prostate cancer cells. Int. J. Oncol..

[71] Zhang X (2017). Phospholipid Phosphatase 4 promotes proliferation and tumorigenesis, and activates Ca2 + -permeable Cationic Channel in lung carcinoma cells. Mol. Cancer.

[72] Zhang X (2017). Thymosin beta 10 is a key regulator of tumorigenesis and metastasis and a novel serum marker in breast cancer. Breast Cancer Res:.

