# Supplementary material for: Transcriptional downregulation of miR-133b by REST promotes prostate cancer metastasis to bone via activating TGF-β signaling
Source: Cell Death Dis. 2018 Jul 13;9(7):779. doi: 10.1038/s41419-018-0807-3 (PMC6045651; doi:10.1038/s41419-018-0807-3)
Supplement: Supplementary file 1 — Supplementary Table 1 [file 41419_2018_807_MOESM1_ESM.docx]

**Supplementary Table 1. The relationship between miR-133b expression level and clinical pathological characteristics in 202 patients with prostate adenocarcinoma.**

| Parameters | Number of cases | | PLPP4 IHC expression | | P values |
| --- | --- | --- | --- | --- | --- |
|  |  |  | Low | High |  |
| Histologic | |  |  |  |  |
| Acinar Type | | 198 | 98 | 100 | 0.621 |
| Other | | 4 | 3 | 1 |  |
| T classification | |  |  |  |  |
| T1 – T2 | | 64 | 11 | 53 | <0.001* |
| T3 – T4 | | 138 | 90 | 48 |  |
| N classification | |  |  |  |  |
| N0 | | 157 | 64 | 93 | <0.001* |
| N1 | | 45 | 37 | 8 |  |
| M classification | |  |  |  |  |
| M0 | | 185 | 85 | 100 | <0.001* |
| M1 | | 17 | 16 | 1 |  |
| Gleason score | |  |  |  |  |
| ≤7 | | 104 | 25 | 79 | <0.001* |
| >7 | | 98 | 76 | 22 |  |
| ISUP Grade | |  |  |  |  |
| ≤3 | | 104 | 25 | 79 | <0.001* |
| >3 | | 98 | 76 | 22 |  |
| PSA level | |  |  |  |  |
| ≤20 ng/ml | | 132 | 46 | 86 | <0.001* |
| >20 ng/ml | | 70 | 55 | 15 |  |
| Bone scan or CT or MRI results | |  |  |  |  |
| Normal | | 168 | 81 | 87 | 0.003* |
| Bone metastasis | | 9 | 9 | 0 |  |

* ISUP: International Society of Urological Pathology, N/A: Not available, PSA: Prostate-specific antigen.
