# Supplementary material for: Transcriptional downregulation of miR-133b by REST promotes prostate cancer metastasis to bone via activating TGF-β signaling
Source: Cell Death Dis. 2018 Jul 13;9(7):779. doi: 10.1038/s41419-018-0807-3 (PMC6045651; doi:10.1038/s41419-018-0807-3)
Supplement: Supplementary file 2 — Supplementary Table 2 [file 41419_2018_807_MOESM2_ESM.docx]

**Supplementary Table 2. Univariate and multivariate analysis of factors associated with bone metastasis free survival in 176 patients with prostate adenocarcinoma.**

| Characteristics | Univariate analysis | | | Multivariate analysis | |
| --- | --- | --- | --- | --- | --- |
|  | HR (95% CI) | *P* values | HR (95% CI) | | *P* values |
| Age (>62) | 1.20 (0.70-2.04) | 0.514 | 1.17 (0.67-2.05) | | 0.590 |
| T classification | 2.80 (1.53-5.14) | 0.001* | 1.21 (0.57-2.57) | | 0.626 |
| N classification | 3.34 (1.95-5.73) | <0.001* | 2.00 (1.08-3.69) | | 0.027* |
| Gleason score | 1.83 (1.37-2.44) | <0.001* | 0.59 (0.21-1.67) | | 0.319 |
| ISUP Grade | 1.70 (1.34-2.17) | <0.001* | 1.74 (0.69-4.36) | | 0.239 |
| PSA level  (>20 ng/ml) | 2.58 (1.51-4.41) | 0.001* | 1.52 (0.86-2.69) | | 0.151 |
| miR-133b level | 0.08 (0.03-0.22) | <0.001* | 0.040 (0.06-0.94) | | 0.040* |

* ISUP: International Society of Urological Pathology, HR: hazard ratio, CI: confidence interval, PSA: Prostate-specific antigen.
