# Supplementary material for: Transcriptional downregulation of miR-133b by REST promotes prostate cancer metastasis to bone via activating TGF-β signaling
Source: Cell Death Dis. 2018 Jul 13;9(7):779. doi: 10.1038/s41419-018-0807-3 (PMC6045651; doi:10.1038/s41419-018-0807-3)
Supplement: Supplementary file 3 — Supplementary Table 3 [file 41419_2018_807_MOESM3_ESM.docx]

**Supplementary Table 3. Univariate and multivariate analysis of factors associated with overall survival in 202 patients with prostate adenocarcinoma.**

| Characteristics | Univariate analysis | | | Multivariate analysis | |
| --- | --- | --- | --- | --- | --- |
|  | HR (95% CI) | *P* values | HR (95% CI) | | *P* values |
| Age | 1.51 (1.31-1.73) | <0.001* | 1.70 (1.40-2.06) | | <0.001* |
| T classification | 1.512 (0.50-4.60) | 0.463 | 2.52 (0.72-8.83) | | 0.149 |
| N classification | 1.04 (0.29-3.73) | 0.953 | 2.13 (0.38-12.14) | | 0.393 |
| M classification | 3.29 (0.92-11.80) | 0.067 | 6.83 (1.50-31.15) | | 0.013 |
| Gleason score | 1.19 (0.70-2.03) | 0.511 | 1.60 (0.13-19.34) | | 0.713 |
| ISUP Grade | 1.10 (0.74-1.62) | 0.203 | 0.58 (0.08-3.96) | | 0.574 |
| PSA level (>20 ng/ml) | 0.99 (0.94-1.04) | 0.574 | 2.63 (0.52-13.32) | | 0.243 |
| miR-133b level | 1.38 (0.38-5.00) | 0.624 | 6.90 (0.72-66.60) | | 0.095 |

* ISUP: International Society of Urological Pathology, HR: hazard ratio, CI: confidence interval, PSA: Prostate-specific antigen.
