# Supplementary material for: Transcriptional downregulation of miR-133b by REST promotes prostate cancer metastasis to bone via activating TGF-β signaling
Source: Cell Death Dis. 2018 Jul 13;9(7):779. doi: 10.1038/s41419-018-0807-3 (PMC6045651; doi:10.1038/s41419-018-0807-3)
Supplement: Supplementary file 4 — Supplemental Table 4 [file 41419_2018_807_MOESM4_ESM.docx]

**Supplementary Table 4. A list of primers used in the reactions for clone PCR.**

| Gene | Sequence (5` – 3`) |
| --- | --- |
| miR-133b-clone-F | GGCAAGCTCCTGGCATTTGAC |
| miR-133b-clone-R | GTTGGCACAAACTCCATCCTC |
| TGFBRI-3`UTR-1702-F | CAGGGTCAGAGTAACCCATACAG |
| TGFBRI-3`UTR-2736-R | GGATGGACCAGGGATGTCTATGC |
| TGFBRII-3`UTR-1997-F | GGGTGGGCTGAGAGTTAAAGAC |
| TGFBRII-3`UTR-2529-R | ATGGGAACAGGAGGCAGGATG |
| miR-133b-promoter-wt-F | GTTGGCATCGCTGGTTAAACTC |
| miR-133b-promoter-wt-R | GGGACCAAACCTCTCAGGAAGAC |
| REST-clone-F | ATGGCCACCCAGGTAATGGG |
| REST-clone-R | TTACTCCTGCCCTTGAGCTGC |
| CTCF-clone-F | ATGGAAGGTGATGCAGTCGAAG |
| CTCF-clone-R | TCACCGGTCCATCATGCTGAG |
