# Supplementary material for: Transcriptional downregulation of miR-133b by REST promotes prostate cancer metastasis to bone via activating TGF-β signaling
Source: Cell Death Dis. 2018 Jul 13;9(7):779. doi: 10.1038/s41419-018-0807-3 (PMC6045651; doi:10.1038/s41419-018-0807-3)
Supplement: Supplementary file 5 — Supplemental Table 5 [file 41419_2018_807_MOESM5_ESM.docx]

**Supplementary Table 5. A list of primers used in the reactions for real-time RT-PCR.**

| Gene | Sequence (5`– 3`) |
| --- | --- |
| THBS1-F | TGCTATCACAACGGAGTTCAGT |
| THBS1-R | GCAGGACACCTTTTTGCAGATG |
| COL1A1-F | GTGCGATGACGTGATCTGTGA |
| COL1A1-R | CGGTGGTTTCTTGGTCGGT |
| VEGFA-F | AGGGCAGAATCATCACGAAGT |
| VEGFA-R | AGGGTCTCGATTGGATGGCA |
| TGFBRI-F | CACAGAGTGGGAACAAAAAGGT |
| TGFBRI-R | CCAATGGAACATCGTCGAGCA |
| TGFBRII-F | AAGATGACCGCTCTGACATCA |
| TGFBRII-R | CTTATAGACCTCAGCAAAGCGAC |
| PTHRP-F | AAGGTGGAGACGTACAAAGAGC |
| PTHRP-R | CAGAGCGAGTTCGCCGTTT |
| IL-11-F | CGAGCGGACCTACTGTCCTA |
| IL-11-R | GCCCAGTCAAGTGTCAGGTG |
| CTGF-F | TGGAGATTTTGGGAGTACGG |
| CTGF-R | CAGGCTAGAGAAGCAGAGCC |
| NEDD9-F | GACCGTCATAGAGCAGAACAC |
| NEDD9-R | TGCATGGGACCAATCAGAAGC |
| MMP13-F | ACTGAGAGGCTCCGAGAAATG |
| MMP13-R | GAACCCCGCATCTTGGCTT |
| ADAM19-F | ACCCTCAAACCACCACACG |
| ADAM19-R | GCTCACCGTAATCAGTCCTCTA |
| GAPDH-F | TCCTCTGACTTCAACAGCGACAC |
| GAPDH-R | CACCCTGTTGCTGTAGCCAAATTC |
| P1-F | GTGCAAACACTTGCAGCTGAGG |
| P1-R | GTTGCGAGGCTTAATGACAAGG |
| P2-F | TTTCCCACCTGCTCCCATACA |
| P2-R | ACCTGCCCTTTGGGTGATTCC |
| P3-F | TAAATTCAGTGTGATTGTTACCTCCG |
| P3-R | CCCATCTCAGCCTCTCAAAGTG |
| P4-F | CAGGTGTCTGTAATTCCAGCTACTC |
| P4-R | ACCAATACCCATGAACAACGATCTA |
| P5-F | ACAGGCTTAGACAAATGATGCTTGA |
| P5-R | TCTTCTGTTTCTCCAAGGACTGGG |
| MMP3-F | AGTCTTCCAATCCTACTGTTGCT |
| MMP3-R | TCCCCGTCACCTCCAATCC |
| MMP7-F | GAGTGAGCTACAGTGGGAACA |
| MMP7-R | CTATGACGCGGGAGTTTAACAT |
| MMP9-F | AGACCTGGGCAGATTCCAAAC |
| MMP9-R | CGGCAAGTCTTCCGAGTAGT |
| TIMP1-F | AGAGTGTCTGCGGATACTTCC |
| TIMP1-R | CCAACAGTGTAGGTCTTGGTG |
| TIMP2-F | AAGCGGTCAGTGAGAAGGAAG |
| TIMP2-R | GGGGCCGTGTAGATAAACTCTAT |
| TIMP3-F | CAGGTCGCGTCTATGATGGC |
| TIMP3-R | AGGTGATACCGATAGTTCAGCC |
| TIMP4-F | ATCTGTGCAACTACATCGAGC |
| TIMP4-R | CGAGATGGTACAGGGTACTGTG |
