# Supplementary material for: Transcriptional downregulation of miR-133b by REST promotes prostate cancer metastasis to bone via activating TGF-β signaling
Source: Cell Death Dis. 2018 Jul 13;9(7):779. doi: 10.1038/s41419-018-0807-3 (PMC6045651; doi:10.1038/s41419-018-0807-3)
Supplement: Supplementary file 6 — Supplementary Table 6 [file 41419_2018_807_MOESM6_ESM.docx]

**Supplemental Table 6. The basic information of 20 prostate adenocarcinoma patients for miR-133b expression analysis.**

|  | | Cases (n) | Percentage (%) |
| --- | --- | --- | --- |
| Histologic | Acinar Type | 20 | 100.0 |
|  | Other | 0 | 0.0 |
| Age | <62 | 9 | 45.0 |
|  | ≥62 | 11 | 55.0 |
| T classification | T1 | 0 | 0.0 |
|  | T2 | 6 | 30.0 |
|  | T3 | 14 | 70.0 |
|  | T4 | 0 | 0.0 |
| N classification | N0 | 15 | 75.0 |
|  | N1 | 5 | 25.0 |
| M classification | M0 | 19 | 95.0 |
|  | M1 | 1 | 5.0 |
| Gleason score | ≤6 | 1 | 5.0 |
|  | 7 | 10 | 50.0 |
|  | ≥8 | 9 | 45.0 |
| ISUP Grade | 1 | 1 | 5.0 |
|  | 2 | 6 | 30.0 |
|  | 3 | 3 | 15.0 |
|  | 4 | 3 | 15.0 |
|  | 5 | 7 | 35.0 |
| Bone metastasis status | Positive | 1 | 5.0 |
|  | Negative | 19 | 95.0 |

* ISUP: International Society of Urological Pathology.
