# Supplementary material for: Transcriptional downregulation of miR-133b by REST promotes prostate cancer metastasis to bone via activating TGF-β signaling
Source: Cell Death Dis. 2018 Jul 13;9(7):779. doi: 10.1038/s41419-018-0807-3 (PMC6045651; doi:10.1038/s41419-018-0807-3)
Supplement: Supplementary file 7 — Supplementary Table 7 [file 41419_2018_807_MOESM7_ESM.docx]

**Supplemental Table 7. The basic information of 48 patients with benign prostate lesions for miR-133b expression analysis.**

|  | | Cases (n) | Percentage (%) |
| --- | --- | --- | --- |
| Age | <62 | 26 | 54.2 |
|  | ≥62 | 22 | 45.8 |
| Type of diseases | Hyperplasia | 30 | 62.5 |
|  | Prostatitis | 18 | 37.5 |
