# Supplementary material for: Transcriptional downregulation of miR-133b by REST promotes prostate cancer metastasis to bone via activating TGF-β signaling
Source: Cell Death Dis. 2018 Jul 13;9(7):779. doi: 10.1038/s41419-018-0807-3 (PMC6045651; doi:10.1038/s41419-018-0807-3)
Supplement: Supplementary file 8 — Supplementary Table 8 [file 41419_2018_807_MOESM8_ESM.docx]

**Supplemental Table 8. The basic information of 202 prostate adenocarcinoma patients for miR-133b expression analysis.**

|  | | Cases (n) | Percentage (%) |
| --- | --- | --- | --- |
| Histologic | Acinar Type | 198 | 98.0 |
|  | Other | 4 | 2.0 |
| Age | <62 | 101 | 50.0 |
|  | ≥62 | 101 | 50.0 |
| T classification | T1 | 0 | 0.0 |
|  | T2 | 64 | 31.7 |
|  | T3 | 134 | 66.3 |
|  | T4 | 4 | 2.0 |
| N classification | N0 | 157 | 77.7 |
|  | N1 | 45 | 22.3 |
| M classification | M0 | 185 | 91.6 |
|  | M1 | 17 | 8.4 |
| Gleason score | ≤6 | 9 | 4.5 |
|  | 7 | 95 | 47.0 |
|  | ≥8 | 98 | 48.5 |
| ISUP Grade | 1 | 9 | 4.5 |
|  | 2 | 59 | 29.2 |
|  | 3 | 36 | 17.8 |
|  | 4 | 24 | 11.9 |
|  | 5 | 74 | 36.6 |
| PSA level | ≤ 20ng/ml | 132 | 65.3 |
|  | >20 ng/ml | 70 | 34.7 |
| Bone scan or CT or MRI results | Normal | 168 | 83.2 |
|  | Abnormal | 13 | 6.4 |
|  | Bone metastasis | 9 | 4.5 |
|  | Equivocal | 4 | 2.0 |
|  | N/A | 8 | 4.0 |
| The survival follow-up | Alive | 188 | 93.1 |
|  | Death | 14 | 6.9 |
| The bone metastasis follow-up (exclude: BM at first) | Negative | 122 | 60.4 |
|  | Positive | 54 | 26.7 |
|  | N/A | 17 | 8.4 |

* ISUP: International Society of Urological Pathology, N/A: Not available, PSA: Prostate-specific antigen.
** Other histologic cancers included: Duct adenocarcinoma, Squamous cell carcinoma.
