# Supplementary material for: Transcriptional downregulation of miR-133b by REST promotes prostate cancer metastasis to bone via activating TGF-β signaling
Source: Cell Death Dis. 2018 Jul 13;9(7):779. doi: 10.1038/s41419-018-0807-3 (PMC6045651; doi:10.1038/s41419-018-0807-3)
Supplement: Supplementary file 9 — Supplemental Figure legends [file 41419_2018_807_MOESM9_ESM.doc]

**Supplementary Figure legends**

**Supplementary Figure 1. miR-133b is downregulated in PCa tissues. (a)** miR-133b expression levels was decreased in PCa tissues compared with that in benign prostate diseases tissues by analyzing the miRNA sequencing dataset of PCa from GSE36802 (Benign, n = 21; PCa, n = 21). **(b)** miR-133b expression levels was decreased in PCa tissues compared with that in the adjacent tumor tissues (ANT) by analyzing the miRNA sequencing dataset of PCa from GSE76260 (ANT, n = 26; PCa, n = 26).

**Supplementary Figure 2. Low expression of miR-133b correlates with poor clinicopathological characteristics and progression-free survival in PCa patients. (a)** miR-133b expression levels in PCa tissues with different Gleason score as assessed by TCGA. **(b)** miR-133b expression levels in PCa tissues with different tumor volume as assessed by TCGA. **(c)** miR-133b expression levels in PCa tissues with different lymph node metastasis status as assessed by TCGA. **(d)** miR-133b expression levels in PCa tissues with different distant metastasis status as assessed by TCGA. **(e)** Kaplan–Meier analysis of overall survival curves of PCa patients with high miR-133b expression (n = 247) versus low miR-133b expression (n = 247) as assessed by TCGA. The mean survival times: miR-133b-low > 120 months, and miR-133b-high > 120 months. **(f)** Kaplan–Meier analysis of progression-free survival curves of PCa patients with high miR-133b expression (n = 227) versus low miR-133b expression (n = 220) as assessed by TCGA. The mean survival times: miR-133b-low = 50.2 months, and miR-133b-high > 120 months.

**Supplementary Figure 3. (a)** Real-time PCR analysis of miR-133b expression in the indicated PC-3, C4-2B and VCaP cells. Transcript levels were normalized by U6 expression. Error bars represent the mean ± s.d. of three independent experiments. *P < 0.05.

**Supplementary Figure 4. (a)** Silencing miR-133b increased the transcriptional activity based on a TGF-β/Smad-responsive luciferase reporter in VCaP cells. *P < 0.05. **(b)** Real-time PCR analysis of miR-133b expression in TGF-β treated PCa cells. Transcript levels were normalized by U6 expression. Error bars represent the mean ± s.d. of three independent experiments. *P < 0.05. **(c)** Western blot analysis showing that silencing miR-133b increased nuclear translocation of pSMAD3 in VCaP cells. The nuclear protein p84 was used as a nuclear protein marker. **(d)** TGF-β inhibitors SD208 attenuated the stimulatory effects of miR-133b downregulation on invasion and migration abilities in VCaP cells. Error bars represent the mean ± s.d. of three independent experiments. **P* < 0.05.

**Supplementary Figure 5. (a)** Predicted miR-133b targeting sequence and mutant sequences in 3′UTRs of TGFBRI and TGFBRII. **(b)** Individual silencing of TGFBRI and TGFBRII attenuated the stimulatory effects of miR-133b downregulation on invasion and migration abilities in VCaP cells. Error bars represent the mean ± s.d. of three independent experiments. *P < 0.05.

**Supplementary Figure 6.** **(a)** Deletion levels of miR-133b in the PCa dataset from TCGA. (Deletion: n = 31; Not deletion: n = 465). **(b)** The miR-133b expression levels with deletions or without deletions in the PCa dataset from TCGA. (Deletion: n = 29; Not deletion: n = 459).

**Supplementary Figure 7. (a-d)** Overexpression of miR-133b inhibited, while silencing miR-133b enhanced invasion and migration abilities in PCa cells. Error bars represent the mean ± S.D. of three independent experiments. *P < 0.05. **(e)** Real-time PCR analysis of multiple MMP and TIMP families expression, including MMP3, MMP7, MMP9, MMP13, TIMP1, TIMP2, TIMP3 and TIMP4, in the indicated PCa cells. Transcript levels were normalized by GAPDH expression. Error bars represent the mean ± s.d. of three independent experiments. *P < 0.05. **(f)** Western blotting of MMP9, MMP13 and TIMP2 expression in the indicated cells. α-Tubulin served as the loading control.

**Supplementary Figure 8. (a)** Silencing of miR-133b reversed the inhibitory effects of REST downregulation on TGF-β/Smad-responsive luciferase reporter in PCa cells. Error bars represent the mean ± s.d. of three independent experiments. *P < 0.05. **(b-d)** Silencing of miR-133b reversed the inhibitory effects of REST downregulation on invasion and migration abilities in PCa cells. Error bars represent the mean ± s.d. of three independent experiments. *P < 0.05.
