# Supplementary figures and images for: Transcriptional downregulation of miR-133b by REST promotes prostate cancer metastasis to bone via activating TGF-β signaling
Source: Cell Death Dis. 2018 Jul 13;9(7):779. doi: 10.1038/s41419-018-0807-3 (PMC6045651; doi:10.1038/s41419-018-0807-3)

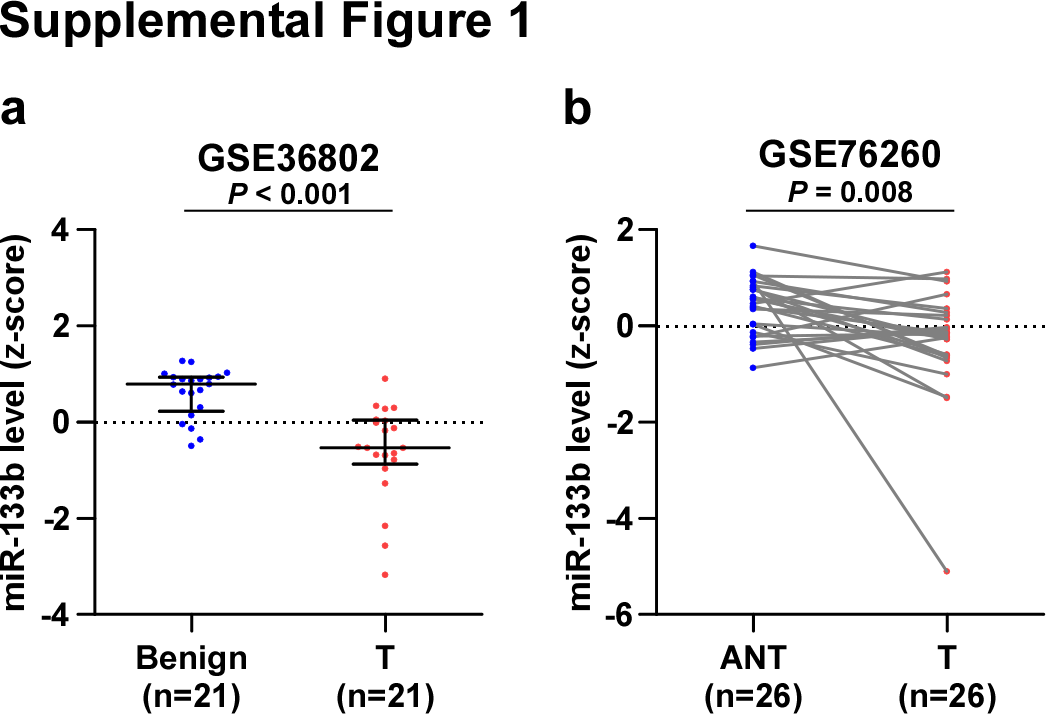

Supplement: Supplementary file 10 — Supplemental Figure 1 [file 41419_2018_807_MOESM10_ESM.tif]

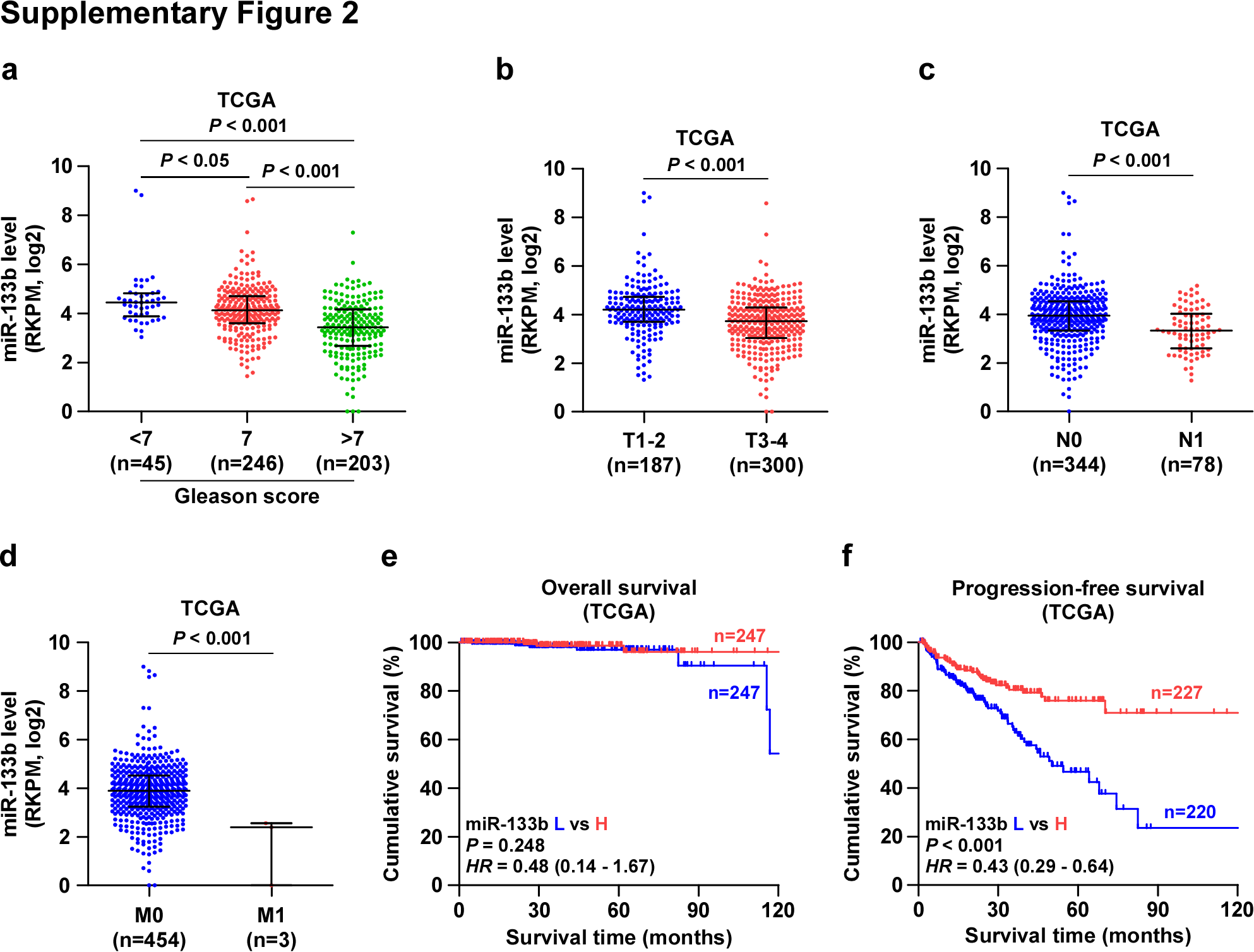

Supplement: Supplementary file 11 — Supplemental Figure 2 [file 41419_2018_807_MOESM11_ESM.tif]

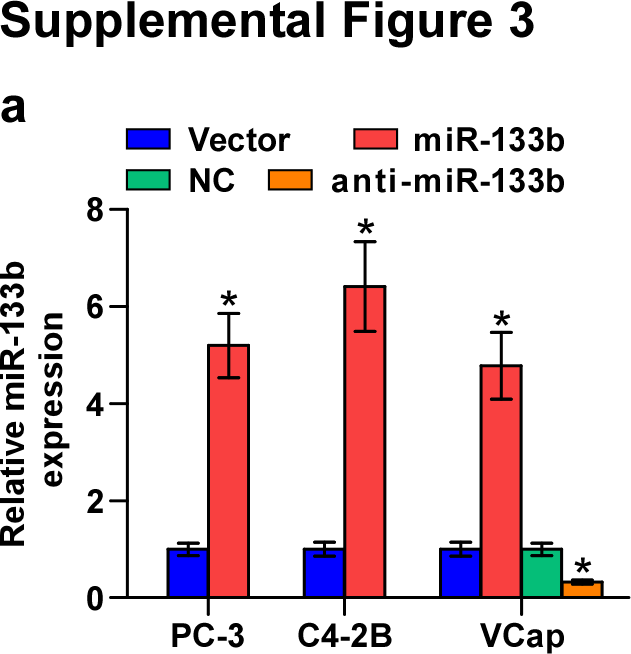

Supplement: Supplementary file 12 — Supplemental Figure 3 [file 41419_2018_807_MOESM12_ESM.tif]

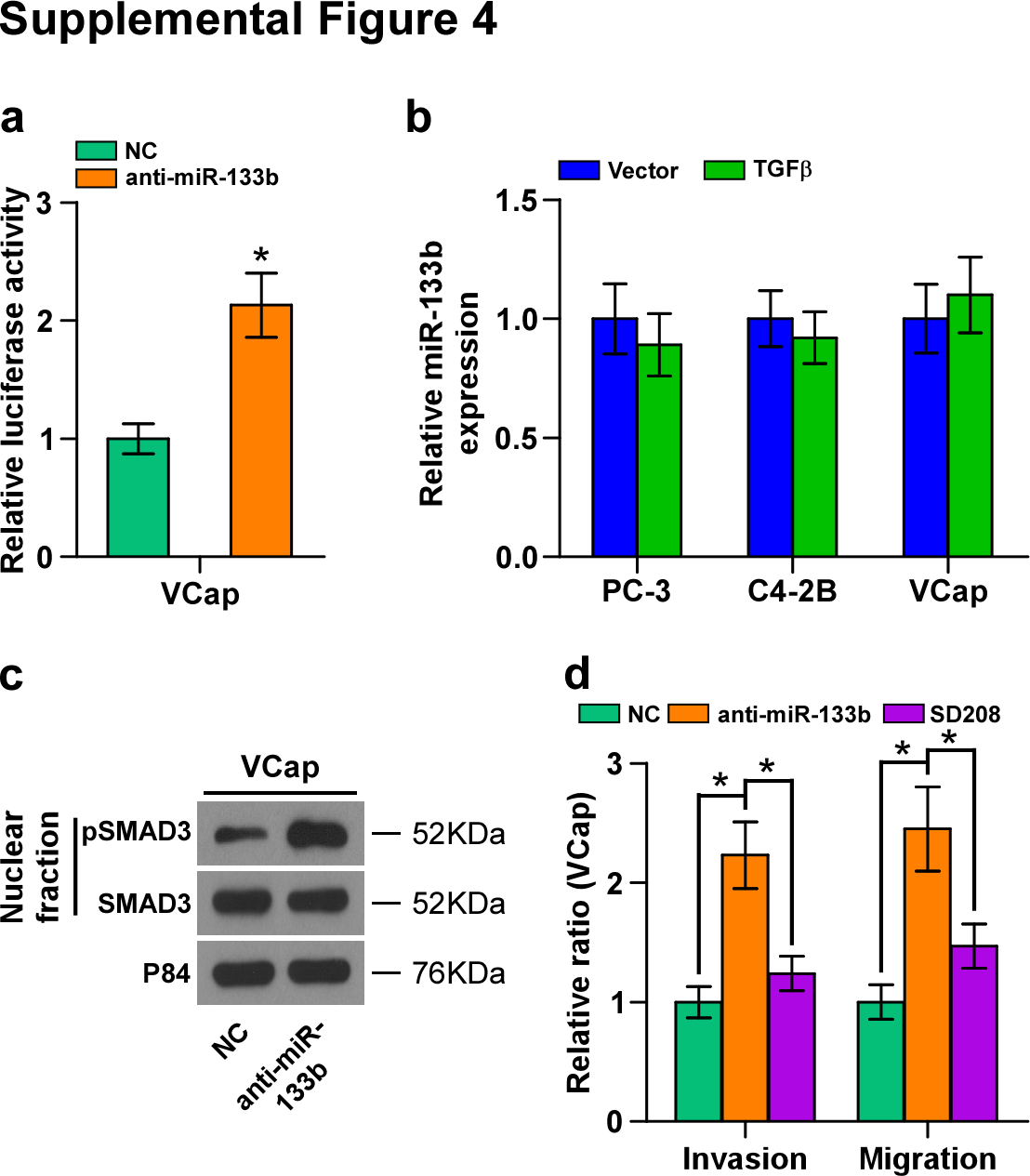

Supplement: Supplementary file 13 — Supplemental Figure 4 [file 41419_2018_807_MOESM13_ESM.tif]

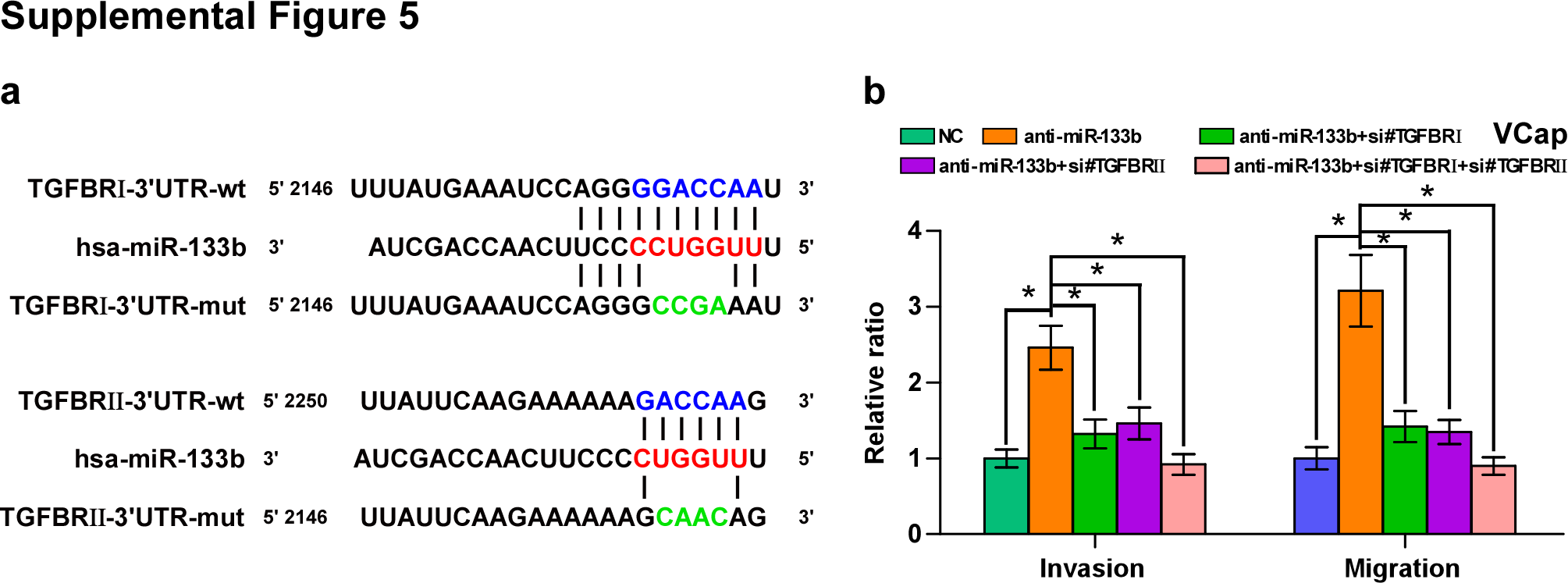

Supplement: Supplementary file 14 — Supplemental Figure 5 [file 41419_2018_807_MOESM14_ESM.tif]

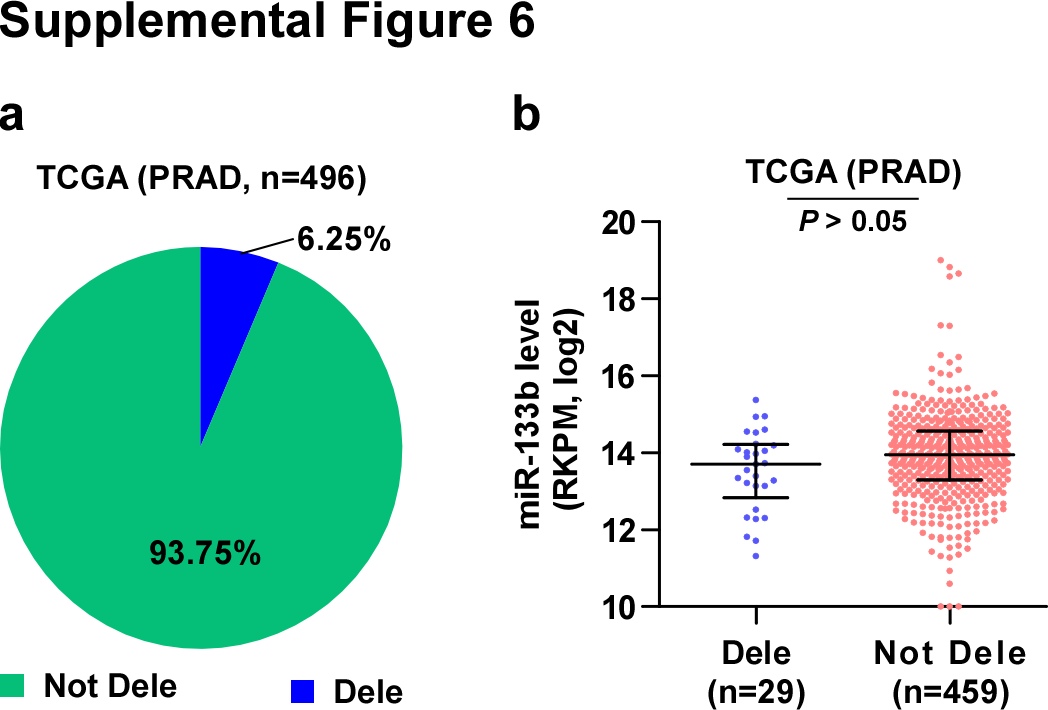

Supplement: Supplementary file 15 — Supplemental Figure 6 [file 41419_2018_807_MOESM15_ESM.tif]

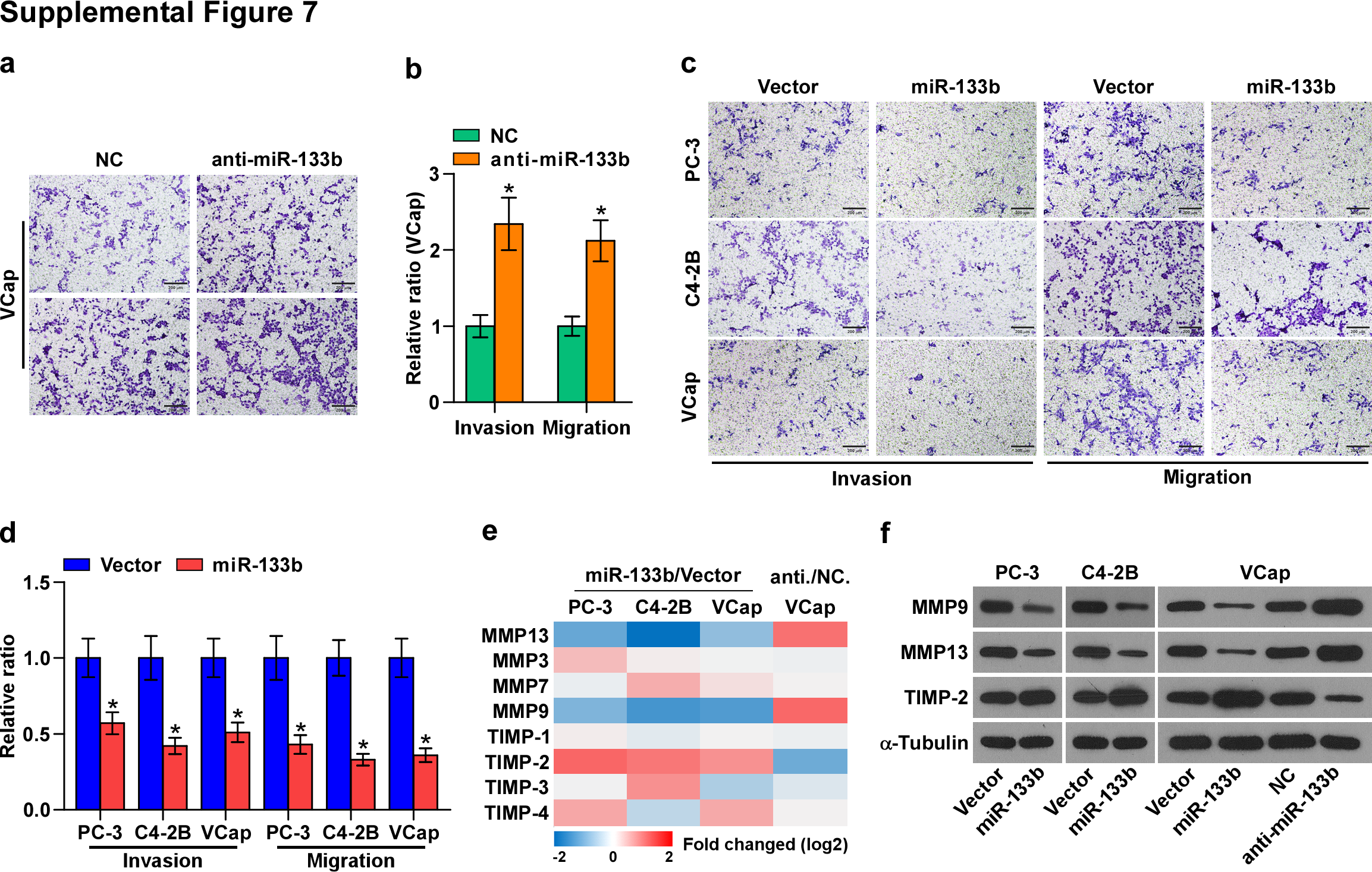

Supplement: Supplementary file 16 — Supplemental Figure 7 [file 41419_2018_807_MOESM16_ESM.tif]

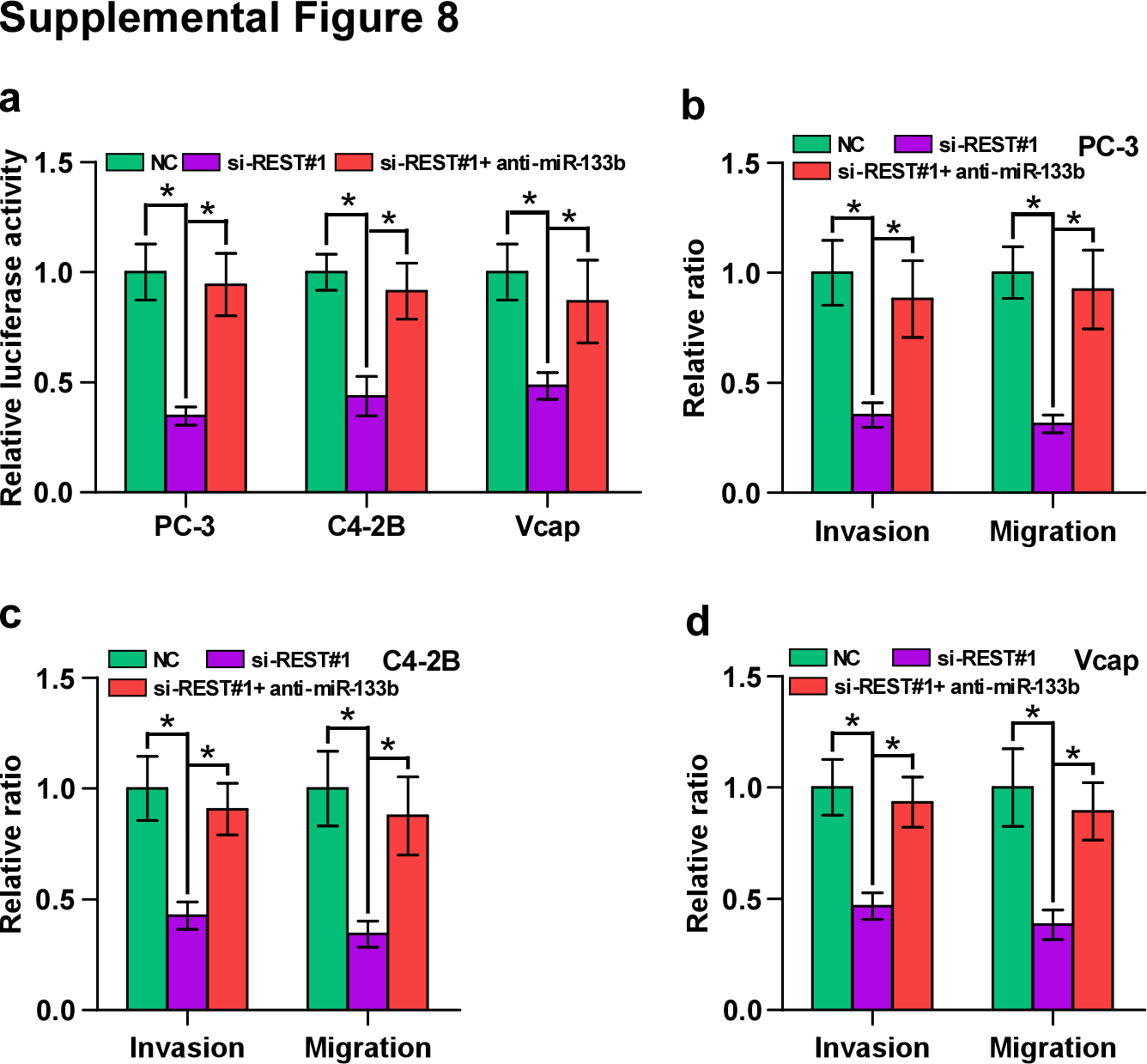

Supplement: Supplementary file 17 — Supplemental Figure 8 [file 41419_2018_807_MOESM17_ESM.tif]
